# Systematic Pharmacology-Based Strategy to Explore the Molecular Network Mechanism of Modified Taohong Siwu Decoction in the Treatment of Premature Ovarian Failure

**DOI:** 10.1155/2022/3044463

**Published:** 2022-01-21

**Authors:** Xiao Yuan, Wang Xiang, Kailin Yang, Huiping Liu, Guomin Zhang, Qi He, Jiapeng Fan

**Affiliations:** ^1^Hunan University of Chinese Medicine, Changsha, Hunan, China; ^2^Harbin Institute of Petroleum, Harbin, Heilongjiang Province, China

## Abstract

**Objective:**

To explore the molecular network mechanism of modified Taohong Siwu Decoction (MTHSWD) to interfere with premature ovarian failure based on systematic pharmacological strategy.

**Methods:**

The network pharmacology strategy was used to explore the potential mechanism of MTHSWD intervention in POF, and then it was verified through animal experiments. Mouse zona pellucida 3 was used as an antigen to subcutaneously immunize BALB/c female mice to establish an immune POF model. Mice were divided into MTHSWD low-, medium-, and high-dose groups, positive control group, model group, and normal group. After 30 days of drug intervention, ovarian tissue was taken for pathological hematoxylin-eosin (HE) staining, and immunohistochemical methods were used to detect the expression of TGF-*β*1 and TGF-*β*RII and Smad2/3 protein expression in follicular wall granular cells and ovarian tissue, respectively.

**Results:**

Network pharmacology studies have shown that MTHSWD may interfere with the TGF-*β* signaling pathway. Animal experimental research shows that, compared with the model group, the number of ovarian mature follicles in the MTHSWD groups and the positive group was significantly increased, and the number of atresia follicles decreased. Immunohistochemistry showed that, compared with the control group, the expression of TGF-*β*1, TGF-*β*RII, and Smad2/3 in the follicular wall granulosa cells and ovarian tissues of MTHSWD groups was significantly higher than that of the model group (*P* < 0.05).

**Conclusion:**

MTHSWD may improve the ovarian function of POF mice by upregulating the protein expression of granulosa cells TGF-*β*1, TGF-*β*RII, and Smad2/3.

## 1. Introduction

Premature ovarian failure (POF) refers to a disease that causes amenorrhea, infertility, menopause, and genitourinary symptoms before the age of 40 due to ovarian failure [1]. Epidemiological studies have shown that the incidence rate in women is about 1% [1, 2]. Hormone detection indicators showed that it has hypogonadotropic hypogonadism [3]. At present, hormone replacement therapy (HRT) is the most popular choice for women with POF to get rid of menopausal syndrome [4]. However, HRT has its own indications and contraindications [5]. For example, unexplained vaginal bleeding, acute liver injury, liver insufficiency, vascular embolism, and breast cancer are contraindications to HRT [6, 7]. In alternative medicine, ancient Chinese medicine has accumulated a lot of clinical experience. With the increase of clinical evidence, TCM has shown a good effect in the treatment of POF [8–10]. The systematic review and meta-analysis of Bushen Huoxue (nourishing the kidney and promoting blood circulation) Chinese herbal medicine for POF showed that compared with the western medicine group, Chinese medicine may improve the total effective rate, menstrual improvement rate, symptom score improvement, and so on, and the incidence of adverse reactions is low [11, 12]. Nourishing the kidney and promoting blood circulation, Chinese medicine may also reduce the level of follicle-stimulating hormone, increase the level of estrogen, reduce clinical symptoms, promote the growth of antral follicles, increase the volume of the ovary, increase the blood flow speed and blood flow pulsation of the ovary, and reduce the blood flow resistance of the ovary. It may also reduce the level of osteocalcin and serum alkaline phosphatase in the body, increase the level of calcitonin, delay the occurrence of osteoporosis, reduce triglycerides and total cholesterol, and reduce the risk of cardiovascular and cerebrovascular diseases. It may also increase the level of CD3+ and CD4+ lymphocytes, improve the body's immunity, and reduce the recurrence rate after drug withdrawal [13–16].

Siwu Decoction [17] was first published in the “Secret Recipe of Xianshou Li Shang” by Lin Taoist in the Tang Dynasty. It was used to treat traumatic diseases, iron beating injuries, blood loss, and blood stasis. In the Song Dynasty, “Tai Ping Hui Min He Ji Ju Fang” began to develop Siwu Decoction into a special prescription for the treatment of gynecological diseases, enriching blood, promoting blood circulation, and regulating menstruation [18, 19]. Since then, physicians of the past generations have elaborated and exerted the use of Siwu Decoction in the treatment of gynecological diseases. They believed that the effect of Siwu Decoction was to enrich blood, promote blood circulation, regulate menstruation, and treat many diseases caused by blood deficiency and blood addiction [19]. On this basis, the addition and subtraction changes have formed many Siwu Decoctions as the core to treat women's abdominal pain during menstruation, that is, Siwu Decoction prescriptions for gynecological blood stasis dysmenorrhea [20]. Among them, Taohong Siwu Decoction is the main representative prescription of nourishing blood and promoting blood circulation in Siwu Decoctions. Recent studies have shown that Modified Taohong Siwu Decoction (MTHSWD) combined with HRT may significantly improve ovarian function and improve the clinical efficacy of the treatment of POF [21]. MTHSWD can improve the symptoms of late menstruation, decreased menstrual flow, irritability, and vaginal dryness in patients with decreased ovarian reserve, reduce FSH levels, improve ovarian blood supply, and increase the number of antral follicles [22]. In addition, the treatment of infertility patients with Shou Tai Wan combined with Taohong Siwu Decoction can effectively improve the quality of pregnancy and improve the immune function and ovarian function of the body [23]. Further studies have shown that Siwu Decoction can improve ovarian reserve and improve follicular dysplasia [24, 25].

However, the molecular biological network mechanism of MTHSWD in the treatment of POF is still not clear. Network pharmacology combines the ideas of systems biology and multidirectional pharmacology. It analyzes the mechanism of action of the effective ingredients of drugs by constructing a complex network within “component targets-pathways-disease,” which turns pharmacological research from the traditional research concept of finding a single target to the network comprehensive analysis thinking [26, 27]. In network pharmacology research, the same disease can be regulated by different genes at different stages of development, and some genes can also play a central regulatory role in multiple diseases. This coincides with the traditional Chinese medicine theory of “different treatment of the same disease” and “treatment of the same disease at the same time” [28, 29]. Therefore, this study hopes to explore the molecular biological network effect and the basis of pharmacodynamic active ingredients of MTHSWD in the treatment of POF by combining systemic pharmacology and experimental pharmacology strategies, so as to provide a scientific basis for the clinical application of MTHSWD.

## 2. Materials and Methods

### 2.1. Potential Compounds and Targets of MTHSWD and POF Gene Collection

The potential components and targets of MTHSWD were searched from TCMSP (https://tcmsp-e.com/) [30] according to the pharmacokinetic parameters of chemical components (absorption, distribution, metabolism, and excretion (ADME)). The standard was oral bioavailability (OB) ≥ 30%, Caco-2 parameter > −0.4, and drug-like activity (DL) ≥ 0.18 [30]. The POF genes were collected to search the Online Mendelian Inheritance in Man (OMIM, http://omim.org/) [31] and GeneCards (http://www.genecards.org/) [32]. The official gene symbol of MTHSWD potential targets and POF genes were collected from UniProt KB (https://www.uniprot.org/uniprot/), with the species restricted to human (Table S1 and Table S2).

### 2.2. Network Construction and Analysis Methods

The String database (https://string-db.org/) was utilized to collect the PPI data of MTHSWD targets and POF genes [33]. The Cytoscape 3.7.2 was utilized to construct and analyze the MTHSWD-POF PPI network [34]. The DAVID ver 6.8 (https://david.ncifcrf.gov/) was utilized to perform gene ontology (GO) enrichment and Kyoto Encyclopedia of Genes and Genomes (KEGG) pathway analysis [35].

### 2.3. Experimental Materials

#### 2.3.1. Experimental Animal

Sixty-seven healthy female BALB/c mice (SYXK (Xiang) 2013–0005), aged 7–8 weeks and weighing 20–22 g, were selected. The vaginal exfoliated cell smear showed that the estrus cycle was normal. Mice were kept in a clean environment, room temperature 18∼22°C, relative humidity 40%∼60%, and light for 12 h.

#### 2.3.2. Experimental Drugs

MTHSWD is composed of *Rehmanniae Radix Praeparata* (Di Huang) 15 g, *Polygonatum sibiricum* Red. (Huang Jing) 12 g, *Cornus officinalis* Sieb. et Zucc. (Shan Zhu Yu) 12 g, *Lycium barbarum* L. (Gou Qi Zi) 12 g, *Angelica sinensis* (Oliv.) Diels (Dang Gui) 15 g, *Paeonia lactiflora* Pall. (Bai Shao) 12 g, *Ligusticum chuanxiong* Hort. (Chuan Xiong) 9 g, *Salvia miltiorrhiza* Bge. (Dan Shen) 12 g, and *Prunus persica* (L.). Batsch (Tao Ren) 9 g. Those medical materials were purchased from Pharmacy Department of the First Affiliated Hospital of Hunan University of Chinese Medicine. The Department of Pharmacy, the First Affiliated Hospital of Hunan University of Chinese Medicine, identified, screened, washed, processed, sliced, dried, and crushed the source and variety of the same batch of Chinese medicinal materials. The batch number was 20150701. Estradiol valerate tablets were obtained from Bayer Healthcare Co., Ltd., National Medicine Standard J20130009.

#### 2.3.3. Instruments and Reagents

Mouse zona pellucida polypeptide solution and the 330–342 amino acid sequence of mouse zona pellucida 3 (ZP3) (NSSSSQFQIHGPR), with an analytical purity of 91.5%, were obtained from Hangzhou Zhongpi Biochemical Co., Ltd. (P00105). Freund's complete adjuvant (F5881) and Freund's incomplete adjuvant (F5506) were obtained from American Sigma company. Rabbit anti-mouse TGF-*β*1, TGF-*β*RII, and Smad2/3 polyclonal antibodies were obtained from Wuhan Boster Bioengineering Co., Ltd. (BA0290, BA0526, BA1395). TRIzol reagent was obtained from Thermo Fisher Technology (China) Co., Ltd. [lot number 267309]. MonScri RTIII All-in-One Mix with sdDNase was obtained from Mona Biotechnology Co., Ltd. (lot number 130449). Universal SYBR qPCR Master Mix Universal Real-Time PCR Kit was obtained from China Biosharp Company (lot number 70090100). Horseradish peroxidase- (HRP-) labeled secondary antibody was obtained from Beijing Zhongshan Jinqiao Biotechnology Co., Ltd. (ZDR-5118). Whole protein extraction kit was obtained from Nanjing KGI Biotechnology Development Co., Ltd. (KGP2100). BCA protein quantitative detection kit was obtained from Shanghai Shenggong Biological Engineering Co., Ltd. (C503031). LEICADMLB2 binocular microscope and LEICARM2255 automatic rotary microtome were obtained from German LEICA company.

### 2.4. Quality Control of MTHSWD

#### 2.4.1. Preparation of Sample

Control solution preparation: morroniside 4.07 mg, loganin 1.16 mg, and paeoniflorin 2.34 mg were accurately weighed into a 10 mL volumetric flask. Then methanol : water (1 : 1) was added, dissolved, and diluted to the mark and filtered through a 0.45 *μ*m filter membrane to make a mixed reference solution.

MTHSWD solution preparation: the medicinal materials of MTHSWD were placed in a round-bottomed flask, 1000 mL of pure water was added, and the mixture was refluxed, extracted for 1.5 h, and filtered. Then, 1000 mL of pure water was added, refluxed for extraction for 1 h, and filtered. The two filtrates were combined and concentrated to 500 mL by rotary evaporation. 2 mL of the concentrated filtrate was added into a 5 mL volumetric flask, and anhydrous methanol was added to the volume. Then, the filtrate was ultrasonically processed (power 300 W, frequency 40 Hz) for 30 min and filtered by suction. The subsequent filtrate was filtered with a 0.45 *μ*m organic microporous filter membrane.

#### 2.4.2. HPLC Condition

Chromatographic column was Hypersil ODS C18 chromatographic column (200 mm × 4.6 mm, 5 *μ*m). Flow rate was 1.0 mL/min. Detection wavelength was 237 nm. Mobile phase was acetonitrile (A)-0.1% phosphoric acid solution (B) gradient elution. Injection volum was 10 *μ*L. Column temperature was 30°C. The contents of those components are morroniside 4.612 ± 0.013 mg/g, loganin 1.291 ± 0.003 mg/g, and paeoniflorin 3.084 ± 0.009 mg/g (Figure S2).

### 2.5. Experimental Methods

#### 2.5.1. Experimental Grouping, Modeling, and Intervention

According to the random number table method, 67 female BALB/c mice were divided into (N) 10 mice as the blank group, and the remaining 57 were prepared for modeling. Add 6 mg of ZP3 transparent polypeptide powder, add 6 mL of double-distilled water to make a solution, and make a 1 : 1 immune reagent with Freund's complete adjuvant; it is formulated with Freund's incomplete adjuvant at a ratio of 1 : 1 to prepare immune enhancement reagents (both in the form of porous white viscous oil). Mice were given 0.15 mL of immune reagent injected into the soles of both hind feet and subcutaneously in the abdominal cavity. 14 days later, 0.15 mL of immunoenhancing reagent was injected again into the soles of both hind feet and subcutaneously in the abdominal cavity to establish an immune POF model. Mice in the blank group was injected with 0.15 mL of normal saline into the same area. Beginning on the 8th day after modeling, all mice were subjected to cervical mucus smears and HE staining of vaginal exfoliated cells at 9 : 00 every morning. The observation lasted for 12 days and the estrus cycle of the mice was checked. The results showed that 5 mice still did not have any estrous cycle disorder and were eliminated.

In the remaining 52 mice, 2 were randomly selected to observe follicular morphology to confirm the POF model. The other 50 mice were randomly divided into model group, positive control group, MTHSWD low-dose group, middle-dose group, and high-dose group, with 10 mice in each group. The dosages of MTHSWD low-, medium-, and high-dose groups were 0.54 g, 1.08 g, and 2.16 g of crude drug/mL, respectively. The low, medium, and high doses of MTHSWD were converted by 1, 2, and 4 times the adult clinical dose according to the “Equivalent Dose Table for Conversion of Human and Animal Body Surface Areas,” respectively [36, 37]. The positive control group was given 0.03 mg of Estradiol. The intragastric administration was started 1 week after the model was established. Both the blank group and the model group were given 0.3 mL of normal saline. The intervention lasted 30 days and was given by gavage. The mice were weighed every 7 days.

#### 2.5.2. Pathological Observation

The ovaries were fixed in 4% paraformaldehyde solution, dehydrated with gradient alcohol, embedded in paraffin, sectioned, deparaffinized, and stained with the conventional hematoxylin-eosin (HE) method.

#### 2.5.3. Expression of TGF-*β*1, TGF-Β*β*RII, and Smad2/3 Protein in Ovarian Tissue Detected by Immunohistochemistry

The ovaries were fixed in 4% paraformaldehyde solution, dehydrated with gradient alcohol, embedded in paraffin, sectioned, and deparaffinized. Then the expression of TGF-*β*1, TGF-*β*RII, and Smad2/3 protein in ovarian tissue was detected by immunohistochemistry SP two-step method. Then the areas under the microscope were randomly selected and analyzed with Image-Pro Plus 6.0. The cumulative optical density (IOD) and area and mean density were measured according to the standard operation method.

#### 2.5.4. Determination of Smad2, Smad3, and Smad7 mRNA Expression in Ovarian Tissue

The total RNA of mouse ovarian tissue was extracted according to the TRIzol method, and the first-strand cDNA was synthesized by reverse transcription, and Smad2, Smad3, and Smad7 mRNA were detected according to the Real-Time PCR method, and *β*-actin was used as the internal control. The reaction was prepared according to the operating instructions of the kit, and the primer sequence was synthesized by Shenggong Bioengineering (Shanghai) Co., Ltd. The reaction conditions were 95°C predenaturation for 2 min, 95°C denaturation for 15 s, 60°C annealing for 20 s, 72°C extension for 30 s, and 40 cycles of amplification. The 2-△△Ct method was used to analyze mRNA expression levels (Table 1).

### 2.6. Statistical Analysis

The SPSS 21.0 statistical software was used for analysis, and the data were expressed as mean ± standard deviation. Multigroup analysis was performed by single-factor analysis of variance. *P* < 0.05 indicated that the difference was statistically significant.

## 3. Results

### 3.1. MTHSWD Potential Targets and POF Targets

**Figure 1:**
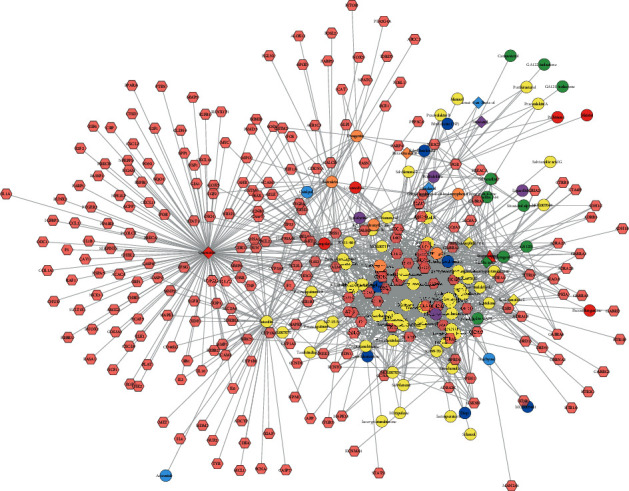
Compound-compound target of MTHSWD (red, orange, yellow, green, blue, indigo, and purple circles stand for components of *Paeonia lactiflora* Pall., *Polygonatum sibiricum* Red., *Salvia miltiorrhiza* Bge., *Prunus persica* (L.) Batsch*, Rehmanniae Radix Praeparata*, *Cornus officinalis* Sieb. et Zucc., and *Ligusticum chuanxiong* Hort., respectively. Red diamond stands for component of *Lycium barbarum* L. Orange diamond stands for common component of *Polygonatum sibiricum* Red.*, Prunus persica* (L.) Batsch*, Rehmanniae Radix Praeparata, Cornus officinalis* Sieb. et Zucc., and *Angelica sinensis* (Oliv.) Diels. Yellow diamond stands for the common components of *Angelica sinensis* (Oliv.) Diels and *Ligusticum chuanxiong* Hort. Green diamond stands for the common components of *Angelica sinensis* (Oliv.) Diels and *Lycium barbarum* L. Blue diamond stands for the common components of *Ligusticum chuanxiong* Hort. and *Cornus officinalis* Sieb. et Zucc. Indigo diamond stands for the common components of *Cornus officinalis* Sieb. et Zucc. and *Salvia miltiorrhiza* Bge. Purple diamond stands for the common components of *Paeonia lactiflora* Pall.*, Polygonatum sibiricum* Red., *Cornus officinalis* Sieb. et Zucc., and *Ligusticum chuanxiong* Hort. Red triangle stands for the common components of *Rehmanniae Radix Praeparata, Cornus officinalis* Sieb. et Zucc., and *Angelica sinensis* (Oliv.) Diels.).

A total of 247 MTHSWD potential targets were obtained and 754 POF genes were collected from OMIM and GeneCards. The relationship among compounds and targets of MTHSWD is shown in Figure 1. This network consists of 100 compound nodes, 247 potential target nodes, and 1527 edges. In this network, the targets near the center can be regulated by more components than targets near the periphery.

### 3.2. MTHSWD-POF PPI Network Analysis

**Figure 2:**
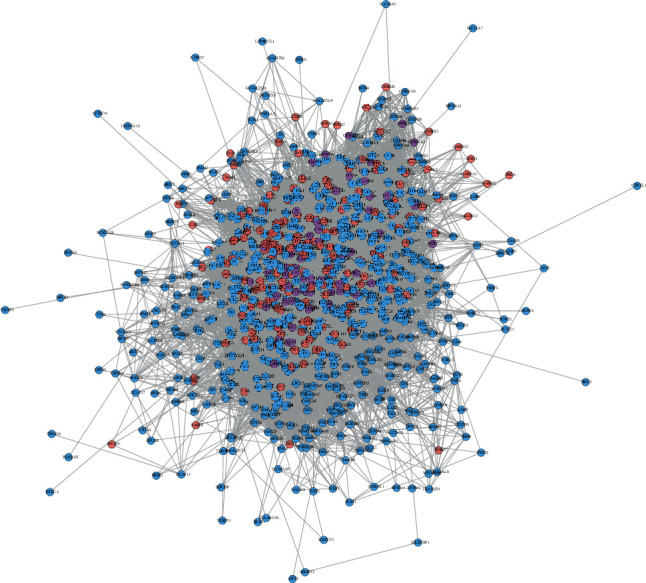
MTHSWD-POF PPI network (blue, pink, and purple circles stand for POF gene, MTHSWD target, MTHSWD-POF target, resp.).

The MTHSWD potential targets, POF genes, and the PPI data were input into Cytoscape 3.7.2 to construct MTHSWD-POF PPI network. This network consists of 823 nodes (578 POF gene nodes, 183 MTHSWD potential target nodes, and 62 MTHSWD-POF target nodes) and 19442 edges. The targets are arranged according to degree from large to small, and the top 20 targets can be divided into 3 categories: (1) MTHSWD potential targets: JUN, EGFR, IL1B, EGF, HIF1A, and FOS; (2) POF genes: ACTB, ALB, INS, and IGF1; (3) MTHSWD-POF targets: TP53, AKT1, IL6, MYC, TNF, ESR1, VEGFA, STAT3, CASP3, and PTEN (Figure 2).

### 3.3. Enrichment Analysis of MTHSWD-POF PPI Network

#### 3.3.1. Biological Processes of MTHSWD-POF PPI Network

**Figure 3:**
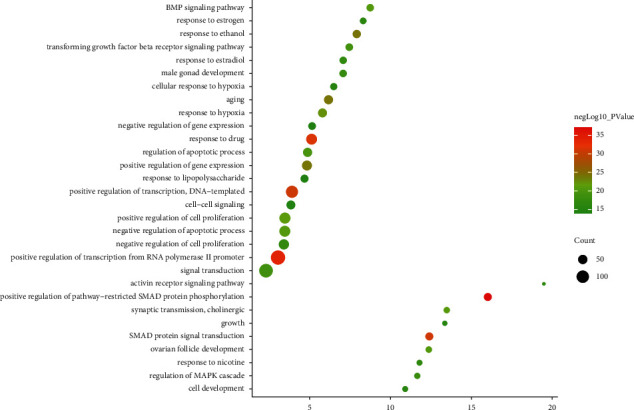
Bubble chart of biological processes (*X*-axis stands for fold enrichment).

The biological processes include positive regulation of pathway-restricted SMAD protein phosphorylation, positive regulation of transcription from RNA polymerase II promoter, response to drug, SMAD protein signal transduction, positive regulation of DNA-templated transcription, aging, response to ethanol, positive regulation of gene expression, response to hypoxia, positive regulation of cell proliferation, cholinergic synaptic transmission BMP signaling pathway, negative regulation of apoptotic process, ovarian follicle development, regulation of apoptotic process, transforming growth factor beta receptor signaling pathway, and signal transduction (Figure 3) (Table S3).

#### 3.3.2. Cell Components of MTHSWD-POF PPI Network

**Figure 4:**
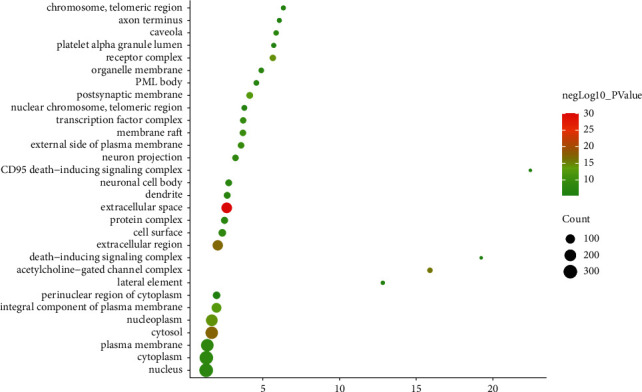
Bubble chart of cell components (*X*-axis stands for fold enrichment).

The cell components include extracellular space, cytosol, extracellular region, acetylcholine-gated channel complex, receptor complex, nucleoplasm, integral component of plasma membrane, postsynaptic membrane, membrane raft, external side of plasma membrane, transcription factor complex, cell surface, plasma membrane, neuron projection, and cytoplasm (Figure 4) (Table S3).

#### 3.3.3. Molecular Function of MTHSWD-POF PPI Network

**Figure 5:**
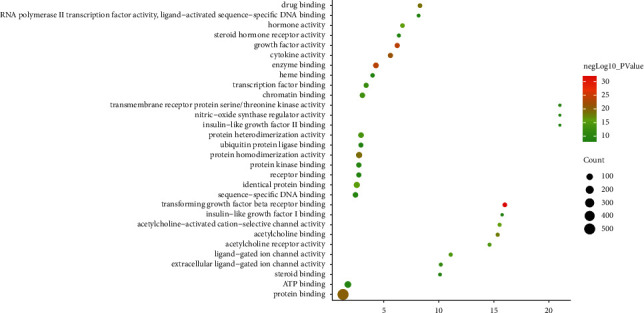
Bubble chart of molecular function (*X*-axis stands for fold enrichment).

The molecular function includes transforming growth factor beta receptor binding, growth factor activity, enzyme binding, cytokine activity, protein binding, protein homodimerization activity, drug binding, acetylcholine binding, acetylcholine-activated cation-selective channel activity, hormone activity, identical protein binding, acetylcholine receptor activity, ligand-gated ion channel activity, and protein heterodimerization activity (Figure 5) (Table S3).

#### 3.3.4. Signaling Pathway of MTHSWD-POF PPI Network

**Figure 6:**
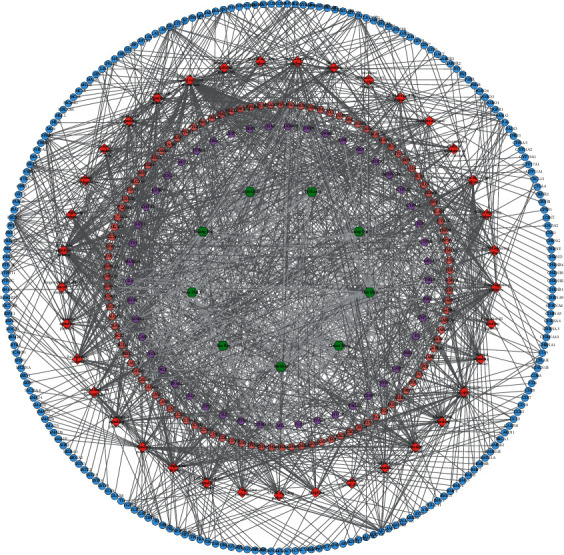
Herb-Target-Signaling pathway network (red diamond stands for signaling pathway. Blue, pink, and purple circles stand for POF gene, MTHSWD target, and MTHSWD-POF target, respectively. Green hexagon stands for herbs).

**Figure 7:**
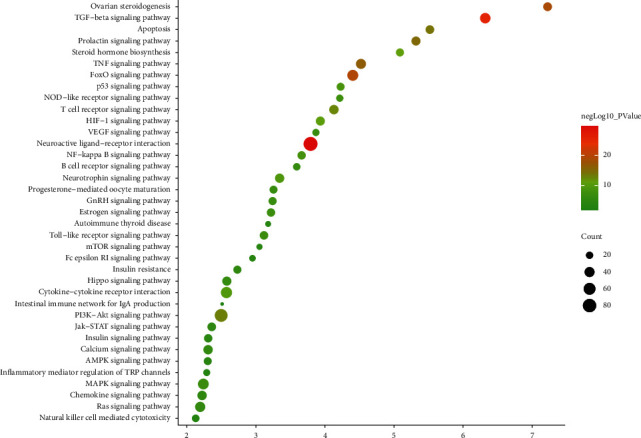
Bubble diagram of signaling pathways (*X*-axis stands for fold enrichment).

**Figure 8:**
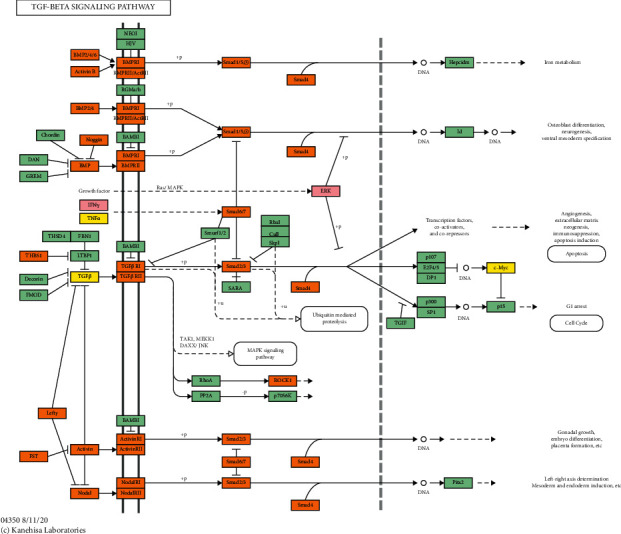
The KEGG Mapper modified from hsa04350 (pink is MTHSWD target, yellow is POF genes, and orange is MTHSWD-POF target).

The signaling pathway includes neuroactive ligand-receptor interaction, TGF-beta signaling pathway, FoxO signaling pathway, ovarian steroidogenesis, TNF signaling pathway, prolactin signaling pathway, apoptosis, PI3K-Akt signaling pathway, T cell receptor signaling pathway, steroid hormone biosynthesis, HIF-1 signaling pathway, cytokine-cytokine receptor interaction, neurotrophin signaling pathway, p53 signaling pathway, NF-kappa B signaling pathway, and NOD-like receptor signaling pathway (Figures 6 and 7). The TGF-beta signaling pathway was shown in Figure 8 (Table S3).

### 3.4. Morphological Changes of Ovarian Tissue

**Figure 9:**
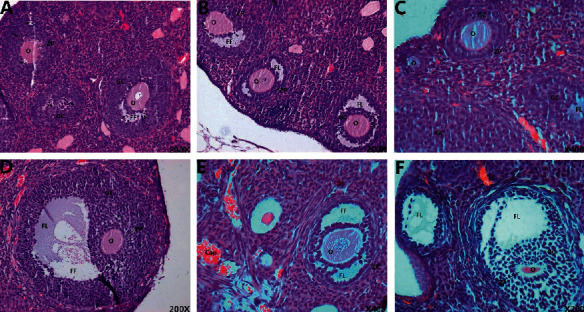
Morphological changes of ovarian tissue (HE staining: 200X). (a) Model group, (b) MTHSWD low-dose group, (c) MTHSWD medium-dose group, (d) MTHSWD high-dose group, (e) positive control group, and (f) blank group. O: oocyte, FL: follicular fluid, FF: follicular cavity, GC: granular cell, ZP: zona pellucida, Cap: blood vessel, and black arrow points to white blood cell.

In model group, the ovarian volume was reduced; a few primary follicles and growing follicles were seen in the ovarian cortex. The number of mature follicles was significantly reduced, the atretic follicles increased, and the secondary follicles were loosely arranged. In MTHSWD medium- and low-dose groups, compared with the model group, the number of primary and mature follicles increased and the number of atretic follicles decreased. In MTHSWD high-dose group and the positive control group, a large number of primary follicles and antral follicles are seen in the ovarian cortex, and there are many near-mature follicles, and the corpus luteum increases and grows well (Figure 9).

### 3.5. Expression of TGF-*β*1, Smad2, and Smad3 mRNA in Ovarian Tissue

**Figure 10:**
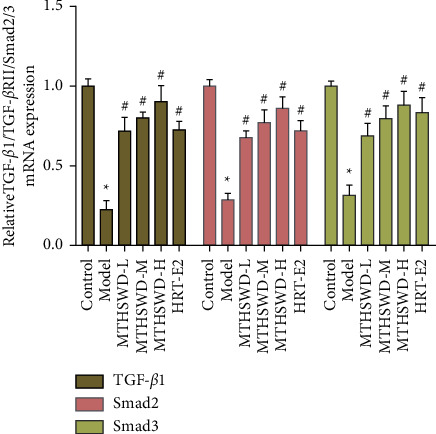
Expression of Smad2, Smad3, and Smad7 mRNA in ovarian tissue (compared with normal group, ^*∗*^*p* < 0.05. Compared with model group, ^#^*P* < 0.05).

Compared with the normal group, the expression levels of TGF-*β*1, Smad2, and Smad3 mRNA in the ovarian tissue of the model group were significantly decreased (*P* < 0.05). Compared with the model group, the expression levels of TGF-*β*1, Smad2, and Smad 3 mRNA in the ovarian tissue of the MTSWD group increased (*P* < 0.05) (Figure 10).

### 3.6. Expression of TGF-*β*1, TGF-Β*β*RII, and Smad2/3 in Granulosa Cells of Ovarian Follicle Wall Detected by Immunohistochemistry

**Figure 11:**
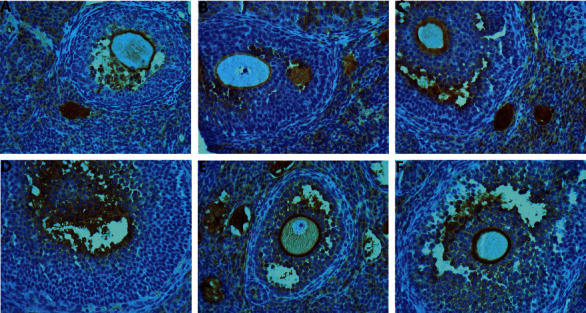
Expression of TGF-*β*1 in granulosa cells (immunohistochemistry: 400X). (a) Model group, (b) MTHSWD low-dose group, (c) MTHSWD medium-dose group, (d) MTHSWD high-dose group, (e) positive control group, and (f) blank group.

**Figure 12:**
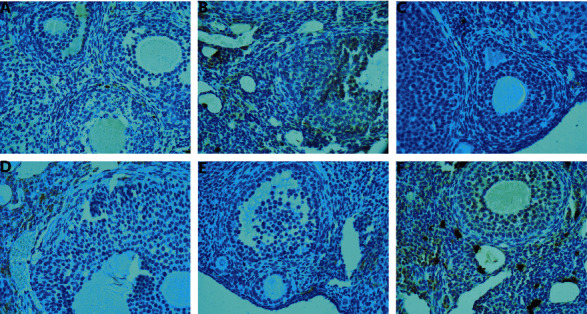
Expression of TGF-*β*RII in granulosa cells (immunohistochemistry: 400X). (a) Model group, (b) MTHSWD low-dose group, (c) MTHSWD medium-dose group, (d) MTHSWD high-dose group, (e) positive control group, and (f) blank group.

**Figure 13:**
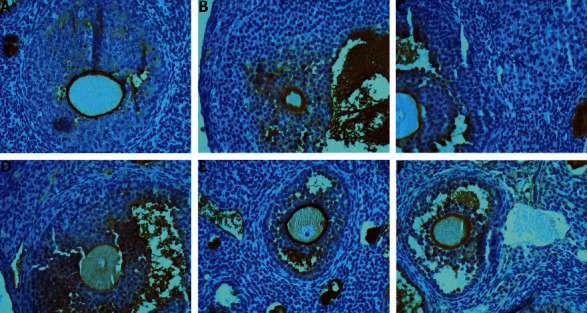
Expression of Smad2/3 in granulosa cells (immunohistochemistry: 400X). (a) Model group, (b) MTHSWD low-dose group, (c) MTHSWD medium-dose group, (d) MTHSWD high-dose group, (e) positive control group, and (f) blank group.

In the model group, TGF-*β*1, Smad2/3, and TGF-*β*RII were weakly expressed in the cytoplasm of granulosa cells of antral follicles. In the MTHSWD low-dose and middle-dose groups, TGF-*β*1 and Smad2/3 were strongly expressed in some areas of antral follicles. In the MTHSWD high-dose group, positive control group, and blank group, TGF-*β*1 and Smad2/3 were strongly expressed in most areas of antral follicles. TGF-*β*RII was strongly expressed in some areas of the antral follicles of MTHSWD low-, medium-, and high-dose groups, positive control group, and blank group (Figures 11–13).

**Figure 14:**
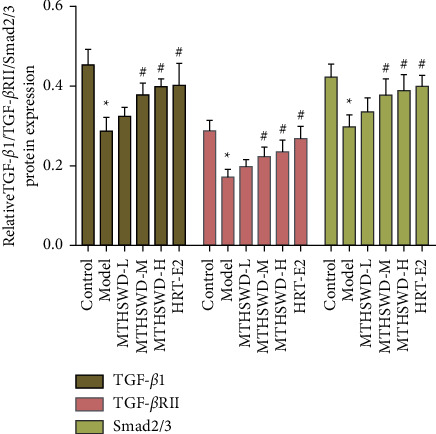
Expression of TGF-*β*1, TGF-*β*RII, and Smad2/3 proteins in granulosa cells (compared with normal group, ^*∗*^*p* < 0.05. Compared with model group, ^#^*P* < 0.05).

Compared with the model group, the expression of TGF-*β*1, TGF-*β*RII, and Smad2/3 in the MTHSWD medium-dose, high-dose group, positive control group, and blank group increased (*P* < 0.05). There was no difference in the expression of TGF-*β*1, TGF-*β*RII, and Smad2/3 in the MTHSWD medium-dose and high-dose groups compared with the positive control group (*P* < 0.05) (Figure 14).

## 4. Discussion

At present, it is more recognized that the factors that cause POF involve genetics, immunity, infection, iatrogenic, psychological, and other factors. In the treatment of POF, hormone replacement or combined use of ovulation induction and assisted reproductive technology is also used for those who have fertility requirements [38]. Previous studies have shown that TCM, which focuses on invigorating the kidney, can reduce the damage of cisplatin to the ovary, promote follicular development, and improve and enhance ovarian function by regulating the content of various hormones in the serum of POF rat models and changing the expression of apoptotic cell-related proteins [39]. Further studies have shown that the Chinese medicine compound for replenishing qi and blood can regulate the rate of estrous cycle disorder in mice with primary ovarian insufficiency, reduce serum LH and FSH levels, and reduce the rate of ovarian granulosa cell apoptosis. It may also increase serum E2 and AMH levels, increase the quality of uterus and ovaries, and increase the expression of TGF-*β*, GDF-9, and BMP15 proteins in ovarian tissue, thereby improving the ovarian function of POF mice [40].

Current studies have shown that mutations in the bone morphogenetic proteins (BMP) gene, which is a member of the transforming growth factor (TGF-*β*) superfamily, will cause serious abnormalities in follicular development and ovulation. The TGF-*β*/Smad signaling pathway involves the growth and development of follicles, the proliferation and apoptosis of granulosa cells and membrane cells, the synthesis of steroid hormones, the maturation of oocytes, ovulation, and luteinization [41, 42]. Smad2 and Smad3 proteins are members of the Smads protein family receptor regulation, and their main function is to participate in signal transduction in the TGF-*β* and activin signaling pathways. At present, siRNA transfection studies have shown that Smad2 and Smad3 are involved in the upregulation of TGF-*β*1 and the production of PGE2, respectively, and then participate in the occurrence and development of ovarian regulation of ovulation [41]. Further studies have shown that Smad2/3 plays an important role in the transition from primordial follicles to primary follicles and the formation of antral follicles [43–45]. The fertility of mice with knocked out Smad2 and Smad3 genes was greatly reduced, and they cannot form normal cumulus expansion and mediate the signal transduction between granulosa cells and oocytes [46]. Researches have showed that Bushen Tiaochong method can effectively increase the expression of TGF-*β*1, Smad3, and P-Smad3 in rat ovarian cells [46]. However, the specific role and mechanism of TGF-*β*/Smad signaling pathway in the granulosa cells of immune POF mice have not been reported yet. The factors that affect follicle development and cell proliferation and differentiation involve multiple signaling pathways, including wnt/*β*-catenin signaling pathway [47], Nodal signaling pathway [48], FGF signaling pathway [49], and BMP/Smads signaling pathway [50]. Smad2 and Smad3 are important factors for maintaining ovarian development and function [51, 52]. A number of studies have shown that the fertility of Smad3 knockout mice is reduced, and the proliferation of granulosa cells is inhibited [53, 54]. Smad2/3 plays an important factor in the granular cells of newly formed primordial follicles [55]. Smad2 and Smad3 knockout mice have greatly reduced fertility [45]. Yang et al. further found that TGF-*β* inhibits the degradation of CyclinD2 through Smad2 and Smad3, activates CDK4, and promotes the synthesis of granular cell DNA [56]. This study found that, compared with the blank group, the protein expression of granulosa cells TGF-*β*1, TGF-*β*RII, and Smad2/3 in the mouse follicles of the model group was significantly reduced, and the follicular atresia was significantly increased. After MTHSWD intervention, the protein expression of TGF-*β*1, TGF-*β*RII, and Smad2/3 was significantly increased (*P* < 0.05), and the expression of MTHSWD high-dose group was significantly higher than that of MTHSWD low-dose group (*P* < 0.05).

## 5. Conclusion

In summary, this study showed that MTHSWD can significantly promote the transmission of the TGF-*β*1/Smads signaling pathway in POF mice, thereby promoting the proliferation and differentiation of granulosa cells. However, since we only studied the TGF-*β*1 ligand in the TGF-*β* pathway, the changes in the local microenvironment formed by the interaction of various factors within the ovary during the development of POF still need to be clearly elucidated. Meanwhile, whether MTHSWD can enhance the signal transcription of other cytokines by increasing the expression of Smads in the treatment of POF mice remains to be further studied.

## Figures and Tables

**Table 1:** Primer sequence of RCR.

Sequence	Upstream 5′–3′	Downstream 5′–3′
TGF-*β*1	CCAAGGAGACGGAATACAGG	GTGTTGGTTGTAGAGGGCAAG
Smad 2	AGCCGCCCGAAGGGTA	AGACCCACCGGCTGATTTTT
Smad 3	CGAGCTGCCTCTGTGCG	CCATCCAGTGACCTGGGGAT
*β*-Asctin	CGCGAGTACAACCTTCTTGC	CGTCATCCATGGCGAACTGG

## Data Availability

All datasets for this study are included in the manuscript and the supplementary files.

## Supplementary Materials

Supplementary Materials Figure S1: the results of HPLC. Table S1: components and targets of MTHSWD. Table S2: POF genes. Table S3: enrichment analysis of MTHSWD-POF PPI network.
