# Supplementary material for: Systematic Pharmacology-Based Strategy to Explore the Molecular Network Mechanism of Modified Taohong Siwu Decoction in the Treatment of Premature Ovarian Failure
Source: Evid Based Complement Alternat Med. 2022 Jan 21;2022:3044463. doi: 10.1155/2022/3044463 (PMC8799328; doi:10.1155/2022/3044463)
Supplement: Supplementary Materials — Figure S1: the results of HPLC. Table S1: components and targets of MTHSWD. Table S2: POF genes. Table S3: enrichment analysis of MTHSWD-POF PPI network. [file 3044463.f1.zip › 3044463.f1/Table S1.pdf]

Table S1 Component and Targets of MTHSWD

Components	Targets
(+)-catechin	PTGS1
(+)-catechin	ESR1
(+)-catechin	PTGS2
(+)-catechin	HSP90AA1
(+)-catechin	NCOA2
(+)-catechin	RXRA
(+)-catechin	CAT
(+)-catechin	HAS2
(+)-catechin	CALM1
(+)-catechin	CALM2
(+)-catechin	CALM3
(2R)-7-hydroxy-2-(4-hydroxyphenyl)chroman-4-one	PTGS1
(2R)-7-hydroxy-2-(4-hydroxyphenyl)chroman-4-one	ESR1
(2R)-7-hydroxy-2-(4-hydroxyphenyl)chroman-4-one	PTGS2
(2R)-7-hydroxy-2-(4-hydroxyphenyl)chroman-4-one	RXRA
(2R)-7-hydroxy-2-(4-hydroxyphenyl)chroman-4-one	PDE3A
(2R)-7-hydroxy-2-(4-hydroxyphenyl)chroman-4-one	ADRB2
(2R)-7-hydroxy-2-(4-hydroxyphenyl)chroman-4-one	HSP90AA1
(2R)-7-hydroxy-2-(4-hydroxyphenyl)chroman-4-one	PIK3CG
(2R)-7-hydroxy-2-(4-hydroxyphenyl)chroman-4-one	MAOB
(2R)-7-hydroxy-2-(4-hydroxyphenyl)chroman-4-one	PRKACA
(2R)-7-hydroxy-2-(4-hydroxyphenyl)chroman-4-one	PKIA
(2R)-7-hydroxy-2-(4-hydroxyphenyl)chroman-4-one	GABRA1
(2R)-7-hydroxy-2-(4-hydroxyphenyl)chroman-4-one	SLC6A4
(2R)-7-hydroxy-2-(4-hydroxyphenyl)chroman-4-one	CALM1
(2R)-7-hydroxy-2-(4-hydroxyphenyl)chroman-4-one	CALM2
(2R)-7-hydroxy-2-(4-hydroxyphenyl)chroman-4-one	CALM3
1,2,5,6-tetrahydrotanshinone	PTGS1
1,2,5,6-tetrahydrotanshinone	CHRM3
1,2,5,6-tetrahydrotanshinone	CHRM1
1,2,5,6-tetrahydrotanshinone	SCN5A
1,2,5,6-tetrahydrotanshinone	CHRM5
1,2,5,6-tetrahydrotanshinone	PTGS2
1,2,5,6-tetrahydrotanshinone	HTR3A
1,2,5,6-tetrahydrotanshinone	CA2
1,2,5,6-tetrahydrotanshinone	CHRM4
1,2,5,6-tetrahydrotanshinone	RXRA
1,2,5,6-tetrahydrotanshinone	OPRD1
1,2,5,6-tetrahydrotanshinone	PDE3A
1,2,5,6-tetrahydrotanshinone	ADRA1A
1,2,5,6-tetrahydrotanshinone	CHRM2
1,2,5,6-tetrahydrotanshinone	ADRA1B
1,2,5,6-tetrahydrotanshinone	SLC6A3
1,2,5,6-tetrahydrotanshinone	ADRB2
1,2,5,6-tetrahydrotanshinone	ADRA1D
1,2,5,6-tetrahydrotanshinone	TOP2A
1,2,5,6-tetrahydrotanshinone	OPRM1
1,2,5,6-tetrahydrotanshinone	GABRA1
1,2,5,6-tetrahydrotanshinone	CHRNA7
1,2,5,6-tetrahydrotanshinone	NCOA2
1,2,5,6-tetrahydrotanshinone	NCOA1
1,2,5,6-tetrahydrotanshinone	DRD1
1,2,5,6-tetrahydrotanshinone	HTR2A
1,2,5,6-tetrahydrotanshinone	SLC6A4

1,2,5,6-tetrahydrotanshinone	IGHG1
2,3-didehydro GA70	PTGS1
2,3-didehydro GA70	CHRM1
2,3-didehydro GA70	PTGS2
2,3-didehydro GA70	SLC6A2
2,3-didehydro GA70	GABRA1
2,3-didehydro GA70	PRSS1
2,3-didehydro GA70	GRIA2
3-beta-Hydroxymethyllenetanshiquinone	DRD1
3-beta-Hydroxymethyllenetanshiquinone	F2
3-beta-Hydroxymethyllenetanshiquinone	CHRM1
3-beta-Hydroxymethyllenetanshiquinone	PTGS2
3-beta-Hydroxymethyllenetanshiquinone	CA2
3-beta-Hydroxymethyllenetanshiquinone	RXRA
3-beta-Hydroxymethyllenetanshiquinone	OPRD1
3-beta-Hydroxymethyllenetanshiquinone	ACHE
3-beta-Hydroxymethyllenetanshiquinone	ADRA1A
3-beta-Hydroxymethyllenetanshiquinone	ADRB2
3-beta-Hydroxymethyllenetanshiquinone	OPRM1
3-beta-Hydroxymethyllenetanshiquinone	DPP4
3-beta-Hydroxymethyllenetanshiquinone	CHRNA7
3-beta-Hydroxymethyllenetanshiquinone	IGHG1
3-beta-Hydroxymethyllenetanshiquinone	PRSS1
3-beta-Hydroxymethyllenetanshiquinone	NCOA1
3'-Methoxydaidzein	NOS2
3'-Methoxydaidzein	PTGS1
3'-Methoxydaidzein	ESR1
3'-Methoxydaidzein	AR
3'-Methoxydaidzein	PPARG
3'-Methoxydaidzein	PTGS2
3'-Methoxydaidzein	RXRA
3'-Methoxydaidzein	ESR2
3'-Methoxydaidzein	MAPK14
3'-Methoxydaidzein	GSK3B
3'-Methoxydaidzein	CDK2
3'-Methoxydaidzein	CHEK1
3'-Methoxydaidzein	PRSS1
3'-Methoxydaidzein	PIM1
3'-Methoxydaidzein	CCNA2
3'-Methoxydaidzein	NCOA1
3'-Methoxydaidzein	CALM1
3'-Methoxydaidzein	CALM2
3'-Methoxydaidzein	CALM3
3α-hydroxytanshinone IIA	F2
3α-hydroxytanshinone IIA	CHRM1
3α-hydroxytanshinone IIA	SCN5A
3α-hydroxytanshinone IIA	CHRM5
3α-hydroxytanshinone IIA	PTGS2
3α-hydroxytanshinone IIA	OPRD1
3α-hydroxytanshinone IIA	ACHE
3α-hydroxytanshinone IIA	ADRB2
3α-hydroxytanshinone IIA	OPRM1
3α-hydroxytanshinone IIA	DPP4
3α-hydroxytanshinone IIA	CHRNA7
3α-hydroxytanshinone IIA	PRSS1
3α-hydroxytanshinone IIA	NCOA1

4',5-Dihydroxyflavone	PTGS1
4',5-Dihydroxyflavone	AR
4',5-Dihydroxyflavone	PTGS2
4',5-Dihydroxyflavone	DPP4
4',5-Dihydroxyflavone	HSP90AA1
4',5-Dihydroxyflavone	PIK3CG
4',5-Dihydroxyflavone	MAOB
4',5-Dihydroxyflavone	PRKACA
4-methylenemiltirone	PTGS1
4-methylenemiltirone	DRD1
4-methylenemiltirone	CHRM3
4-methylenemiltirone	CHRM1
4-methylenemiltirone	ESR1
4-methylenemiltirone	AR
4-methylenemiltirone	SCN5A
4-methylenemiltirone	PPARG
4-methylenemiltirone	CHRM5
4-methylenemiltirone	PTGS2
4-methylenemiltirone	NOS3
4-methylenemiltirone	ADRA2A
4-methylenemiltirone	CA2
4-methylenemiltirone	ADRA2C
4-methylenemiltirone	CHRM4
4-methylenemiltirone	RXRA
4-methylenemiltirone	OPRD1
4-methylenemiltirone	HTR2A
4-methylenemiltirone	ADRA1A
4-methylenemiltirone	CHRM2
4-methylenemiltirone	ADRA1B
4-methylenemiltirone	SLC6A3
4-methylenemiltirone	ADRB2
4-methylenemiltirone	ADRA1D
4-methylenemiltirone	TOP2A
4-methylenemiltirone	SLC6A4
4-methylenemiltirone	DRD2
4-methylenemiltirone	OPRM1
4-methylenemiltirone	GABRA1
4-methylenemiltirone	CHRNA7
4-methylenemiltirone	NCOA2
4-methylenemiltirone	NCOA1
537-15-5	F2
537-15-5	ESR1
537-15-5	AR
537-15-5	PPARG
537-15-5	PTGS2
537-15-5	DPP4
537-15-5	PRSS1
537-15-5	CCNA2
87112-49-0	PTGS1
87112-49-0	DRD1
87112-49-0	CHRM3
87112-49-0	CHRM1
87112-49-0	ESR1
87112-49-0	AR
87112-49-0	SCN5A
87112-49-0	PPARG

87112-49-0	CHRM5
87112-49-0	PTGS2
87112-49-0	NOS3
87112-49-0	HTR3A
87112-49-0	CHRM4
87112-49-0	RXRA
87112-49-0	HTR2A
87112-49-0	ADRA1A
87112-49-0	CHRM2
87112-49-0	ADRA1B
87112-49-0	SLC6A3
87112-49-0	ADRB2
87112-49-0	ADRA1D
87112-49-0	SLC6A4
87112-49-0	OPRM1
87112-49-0	GABRA1
87112-49-0	CDK2
87112-49-0	PIK3CG
87112-49-0	CHRNA7
87112-49-0	IGHG1
87112-49-0	PIM1
87112-49-0	CCNA2
87112-49-0	NCOA2
87112-49-0	CALM1
87112-49-0	CALM2
87112-49-0	CALM3
97399-70-7	PTGS1
97399-70-7	DRD1
97399-70-7	CHRM3
97399-70-7	F2
97399-70-7	SCN5A
97399-70-7	CHRM5
97399-70-7	PTGS2
97399-70-7	CA2
97399-70-7	RXRA
97399-70-7	ACHE
97399-70-7	ADRA1A
97399-70-7	ADRB2
97399-70-7	OPRM1
97399-70-7	GABRA1
97399-70-7	DPP4
97399-70-7	PIK3CG
97399-70-7	CHRNA7
97399-70-7	IGHG1
97399-70-7	NCOA1
97411-46-6	F2
97411-46-6	PTGS2
97411-46-6	CA2
97411-46-6	ACHE
97411-46-6	DPP4
97411-46-6	PRSS1
97411-46-6	NCOA1
Acteoside	ICAM1
Baicalein	PTGS1
Baicalein	AR
Baicalein	PTGS2

Baicalein	DPP4
Baicalein	PIK3CG
Baicalein	PDE3A
Baicalein	PRSS1
Baicalein	NCOA2
Baicalein	NCOA1
Baicalein	RELA
Baicalein	AKT1
Baicalein	VEGFA
Baicalein	BCL2
Baicalein	FOS
Baicalein	BAX
Baicalein	MMP9
Baicalein	CASP3
Baicalein	TP53
Baicalein	HIF1A
Baicalein	FOSL1
Baicalein	FOSL2
Baicalein	CDK1
Baicalein	CCNB1
Baicalein	MPO
Baicalein	AHR
Baicalein	IGF2
Baicalein	ALOX12
Baicalein	NFATC1
Baicalein	TDRD7
Baicalein	EGLN1
Baicalein	NOX5
Baicalein	FABP5
Baicalein	APOD
Baicalein	CALM1
Baicalein	CALM2
Baicalein	CALM3
Beta-sitosterol	PGR
Beta-sitosterol	NCOA2
Beta-sitosterol	NR3C2
Butylidenephthalide	PTGS1
Butylidenephthalide	CHRM3
Butylidenephthalide	CHRM1
Butylidenephthalide	ADRB1
Butylidenephthalide	SCN5A
Butylidenephthalide	PTGS2
Butylidenephthalide	ADRA2A
Butylidenephthalide	ADRA2C
Butylidenephthalide	PDE3A
Butylidenephthalide	HTR2A
Butylidenephthalide	SLC6A2
Butylidenephthalide	ADRA1A
Butylidenephthalide	CHRM2
Butylidenephthalide	ADRA2B
Butylidenephthalide	ADRA1B
Butylidenephthalide	SLC6A3
Butylidenephthalide	ADRB2
Butylidenephthalide	SLC6A4
Butylidenephthalide	GABRA1
Butylidenephthalide	MAOB

Butylidenephthalide	MAOA
Butylidenephthalide	RXRA
Butylidenephthalide	PKIA
C09092	CHRM3
C09092	F2
C09092	CHRM1
C09092	SCN5A
C09092	CA2
C09092	ACHE
C09092	ADRA1A
C09092	CHRM2
C09092	ADRA1B
C09092	ADRB2
C09092	ADRA1D
C09092	OPRM1
Campesterol	PGR
Catalpol	DPP4
Catalpol	BCL2
Catalpol	CASP3
Catalpol	SOD1
Cornudentanone	F2
Cornudentanone	PTGS2
Cornudentanone	NCOA2
Cryptotanshinone	PTGS1
Cryptotanshinone	DRD1
Cryptotanshinone	CHRM3
Cryptotanshinone	CHRM1
Cryptotanshinone	SCN5A
Cryptotanshinone	CHRM5
Cryptotanshinone	PTGS2
Cryptotanshinone	CA2
Cryptotanshinone	CHRM4
Cryptotanshinone	OPRD1
Cryptotanshinone	ADRA1A
Cryptotanshinone	CHRM2
Cryptotanshinone	ADRA1B
Cryptotanshinone	ADRB2
Cryptotanshinone	ADRA1D
Cryptotanshinone	TOP2A
Cryptotanshinone	OPRM1
Cryptotanshinone	CHRNA7
Cryptotanshinone	NCOA2
Cryptotanshinone	NCOA1
Cryptotanshinone	PGR
Cryptotanshinone	GABRA1
Cryptotanshinone	RELA
Cryptotanshinone	STAT3
Cryptotanshinone	CCND1
Cryptotanshinone	BCL2L1
Cryptotanshinone	TNF
Cryptotanshinone	APP
Cryptotanshinone	EDN1
Cryptotanshinone	BIRC5
Danshenol A	PTGS1
Danshenol A	KCNH2
Danshenol A	SCN5A

Danshenol A	F10
Danshenol A	PTGS2
Danshenol A	RXRA
Danshenol A	PIK3CG
Danshenol A	NCOA1
Danshenol A	KCNMA1
Danshenol B	PTGS2
Danshenol B	CA2
Danshenol B	PGR
Danshenol B	TOP2A
Danshenol B	OPRM1
Danshenol B	NR3C1
Danshenol B	NCOA1
Danshenspiroketallactone	PTGS1
Danshenspiroketallactone	DRD1
Danshenspiroketallactone	CHRM3
Danshenspiroketallactone	F2
Danshenspiroketallactone	CHRM1
Danshenspiroketallactone	ESR1
Danshenspiroketallactone	SCN5A
Danshenspiroketallactone	CHRM5
Danshenspiroketallactone	PTGS2
Danshenspiroketallactone	CA2
Danshenspiroketallactone	CHRM4
Danshenspiroketallactone	RXRA
Danshenspiroketallactone	ACHE
Danshenspiroketallactone	ADRA1A
Danshenspiroketallactone	CHRM2
Danshenspiroketallactone	ADRA1B
Danshenspiroketallactone	ADRB2
Danshenspiroketallactone	ADRA1D
Danshenspiroketallactone	CHRNA2
Danshenspiroketallactone	SLC6A4
Danshenspiroketallactone	OPRM1
Danshenspiroketallactone	GABRA1
Danshenspiroketallactone	DPP4
Danshenspiroketallactone	CHRNA7
Dan-shexinkum D	NOS2
Dan-shexinkum D	PTGS1
Dan-shexinkum D	F2
Dan-shexinkum D	KCNH2
Dan-shexinkum D	CHRM1
Dan-shexinkum D	ESR1
Dan-shexinkum D	AR
Dan-shexinkum D	SCN5A
Dan-shexinkum D	PPARG
Dan-shexinkum D	F10
Dan-shexinkum D	PTGS2
Dan-shexinkum D	CA2
Dan-shexinkum D	RXRA
Dan-shexinkum D	ACHE
Dan-shexinkum D	ADRA1B
Dan-shexinkum D	ADRB2
Dan-shexinkum D	TOP2A
Dan-shexinkum D	ESR2
Dan-shexinkum D	DPP4

Dan-shexinkum D	GSK3B
Dan-shexinkum D	CDK2
Dan-shexinkum D	CHEK1
Dan-shexinkum D	IGHG1
Dan-shexinkum D	PRSS1
Dan-shexinkum D	PIM1
Dan-shexinkum D	CCNA2
Dan-shexinkum D	NCOA2
Dan-shexinkum D	NCOA1
Dan-shexinkum D	CALM1
Dan-shexinkum D	CALM2
Dan-shexinkum D	CALM3
Dehydrotanshinone IIA	DRD1
Dehydrotanshinone IIA	CHRM3
Dehydrotanshinone IIA	F2
Dehydrotanshinone IIA	CHRM1
Dehydrotanshinone IIA	ESR1
Dehydrotanshinone IIA	AR
Dehydrotanshinone IIA	SCN5A
Dehydrotanshinone IIA	PPARG
Dehydrotanshinone IIA	CHRM5
Dehydrotanshinone IIA	PTGS2
Dehydrotanshinone IIA	CHRM4
Dehydrotanshinone IIA	OPRD1
Dehydrotanshinone IIA	ACHE
Dehydrotanshinone IIA	HTR2A
Dehydrotanshinone IIA	ADRA1A
Dehydrotanshinone IIA	ADRB2
Dehydrotanshinone IIA	OPRM1
Dehydrotanshinone IIA	GABRA1
Dehydrotanshinone IIA	DPP4
Dehydrotanshinone IIA	CHRNA7
Deoxyneocryptotanshinone	PTGS1
Deoxyneocryptotanshinone	DRD1
Deoxyneocryptotanshinone	CHRM3
Deoxyneocryptotanshinone	CHRM1
Deoxyneocryptotanshinone	ESR1
Deoxyneocryptotanshinone	AR
Deoxyneocryptotanshinone	SCN5A
Deoxyneocryptotanshinone	CHRM5
Deoxyneocryptotanshinone	PTGS2
Deoxyneocryptotanshinone	NOS3
Deoxyneocryptotanshinone	CA2
Deoxyneocryptotanshinone	CHRM4
Deoxyneocryptotanshinone	RXRA
Deoxyneocryptotanshinone	OPRD1
Deoxyneocryptotanshinone	ADRA1A
Deoxyneocryptotanshinone	CHRM2
Deoxyneocryptotanshinone	ADRA1B
Deoxyneocryptotanshinone	ADRB2
Deoxyneocryptotanshinone	ADRA1D
Deoxyneocryptotanshinone	TOP2A
Deoxyneocryptotanshinone	OPRM1
Deoxyneocryptotanshinone	GSK3B
Deoxyneocryptotanshinone	CDK2
Deoxyneocryptotanshinone	CHRNA7

Deoxyneocryptotanshinone	IGHG1
Deoxyneocryptotanshinone	PIM1
Deoxyneocryptotanshinone	NCOA2
Deoxyneocryptotanshinone	NCOA1
Dihydrotanshinlactone	NOS2
Dihydrotanshinlactone	PTGS1
Dihydrotanshinlactone	DRD1
Dihydrotanshinlactone	CHRM3
Dihydrotanshinlactone	F2
Dihydrotanshinlactone	CHRM1
Dihydrotanshinlactone	ESR1
Dihydrotanshinlactone	AR
Dihydrotanshinlactone	SCN5A
Dihydrotanshinlactone	PPARG
Dihydrotanshinlactone	CHRM5
Dihydrotanshinlactone	PTGS2
Dihydrotanshinlactone	HTR3A
Dihydrotanshinlactone	CA2
Dihydrotanshinlactone	RXRA
Dihydrotanshinlactone	ACHE
Dihydrotanshinlactone	PDE3A
Dihydrotanshinlactone	HTR2A
Dihydrotanshinlactone	ADRA1A
Dihydrotanshinlactone	ADRA1B
Dihydrotanshinlactone	SLC6A3
Dihydrotanshinlactone	ADRB2
Dihydrotanshinlactone	ADRA1D
Dihydrotanshinlactone	SLC6A4
Dihydrotanshinlactone	OPRM1
Dihydrotanshinlactone	GABRA1
Dihydrotanshinlactone	DPP4
Dihydrotanshinlactone	GSK3B
Dihydrotanshinlactone	PIK3CG
Dihydrotanshinlactone	CHRNA7
Dihydrotanshinlactone	IGHG1
Dihydrotanshinlactone	PRSS1
Dihydrotanshinlactone	PIM1
Dihydrotanshinlactone	CCNA2
Dihydrotanshinone I	PTGS1
Dihydrotanshinone I	SCN5A
Dihydrotanshinone I	PTGS2
Dihydrotanshinone I	HTR3A
Dihydrotanshinone I	RXRA
Dihydrotanshinone I	ADRA1A
Dihydrotanshinone I	ADRA1B
Dihydrotanshinone I	ADRB2
Dihydrotanshinone I	GABRA1
Dihydrotanshinone I	PIK3CG
Dihydrotanshinone I	CHRNA7
Dihydrotanshinone I	IGHG1
Dihydrotanshinone I	NCOA2
Dihydrotanshinone I	NCOA1
Dihydrotanshinone I	CALM1
Dihydrotanshinone I	CALM2
Dihydrotanshinone I	CALM3
Diop	SCN5A

Diop	ADRB2
Diop	CHRM3
Diosgenin	PGR
Diosgenin	NR3C2
Diosgenin	RELA
Diosgenin	AKT1
Diosgenin	VEGFA
Diosgenin	CDKN1A
Diosgenin	TP53
Diosgenin	PTGS2
Diosgenin	FASN
Diosgenin	SOD1
Diosgenin	CAT
Diosgenin	HIF1A
Diosgenin	NR1I2
Diosgenin	PLA2G4A
Diosgenin	ABCC2
Diosgenin	MTOR
Epidanshenspiroketallactone	PTGS1
Epidanshenspiroketallactone	DRD1
Epidanshenspiroketallactone	CHRM3
Epidanshenspiroketallactone	CHRM1
Epidanshenspiroketallactone	ESR1
Epidanshenspiroketallactone	SCN5A
Epidanshenspiroketallactone	CHRM5
Epidanshenspiroketallactone	PTGS2
Epidanshenspiroketallactone	NOS3
Epidanshenspiroketallactone	CHRM4
Epidanshenspiroketallactone	RXRA
Epidanshenspiroketallactone	OPRD1
Epidanshenspiroketallactone	PDE3A
Epidanshenspiroketallactone	HTR2A
Epidanshenspiroketallactone	ADRA1A
Epidanshenspiroketallactone	CHRM2
Epidanshenspiroketallactone	ADRA1B
Epidanshenspiroketallactone	ADRB2
Epidanshenspiroketallactone	ADRA1D
Epidanshenspiroketallactone	SLC6A4
Epidanshenspiroketallactone	OPRM1
Epidanshenspiroketallactone	GABRA1
Epidanshenspiroketallactone	CDK2
Epidanshenspiroketallactone	CHRNA7
Epidanshenspiroketallactone	PIM1
Ethyl linolenate	PTGS1
Ethyl linolenate	NCOA2
Ethyl oleate (NF)	NCOA2
Ferulic acid	PTGS1
Ferulic acid	PTGS2
Ferulic acid	NOS3
Ferulic acid	ADRA2A
Ferulic acid	SLC6A2
Ferulic acid	ADRA1A
Ferulic acid	SLC6A3
Ferulic acid	ADRB2
Ferulic acid	LTA4H
Ferulic acid	MAOB

Ferulic acid	MAOA
Ferulic acid	CTRB1
Ferulic acid	ADRA2B
Ferulic acid	PLAU
Formyltanshinone	F2
Formyltanshinone	AR
Formyltanshinone	PTGS2
Formyltanshinone	RXRA
Formyltanshinone	DPP4
Formyltanshinone	PIK3CG
Formyltanshinone	NCOA1
GA120	CHRM3
GA120	CHRM1
GA120	PTGS2
GA120	GABRA3
GA120	CHRM2
GA120	GABRA1
GA120	CHRNA7
GA120	GABRA6
GA121-isolactone	PGR
GA122-isolactone	PGR
Gibberellin 7	CHRM3
Gibberellin 7	CHRM1
Gibberellin 7	PTGS2
Gibberellin 7	PDE3A
Gibberellin 7	SLC6A3
Gibberellin 7	ADRB2
Gibberellin 7	SLC6A4
Gibberellin A44	NR3C2
Gibberellin A44	GABRA1
Gibberellin A44	GABRA6
Hederagenin	PGR
Hederagenin	NCOA2
Hederagenin	CHRM3
Hederagenin	CHRM1
Hederagenin	GABRA2
Hederagenin	GABRA3
Hederagenin	CHRM2
Hederagenin	ADRA1B
Hederagenin	GABRA1
Hederagenin	GRIA2
Hederagenin	GABRA6
Hederagenin	GABRA5
Hederagenin	IGHG1
Hederagenin	ADH1B
Hederagenin	ADH1C
Hederagenin	PTGS1
Hederagenin	SCN5A
Hederagenin	PTGS2
Hederagenin	RXRA
Hederagenin	PDE3A
Hederagenin	SLC6A2
Hydroxygenkwanin	NOS2
Hydroxygenkwanin	PTGS1
Hydroxygenkwanin	PTGS2
Hydroxygenkwanin	DPP4

Hydroxygenkwanin	PRSS1
Hydroxygenkwanin	NCOA2
Hydroxygenkwanin	PIK3CG
Hydroxygenkwanin	CALM1
Hydroxygenkwanin	CALM2
Hydroxygenkwanin	CALM3
Isocryptotanshinone	NCOA1
Isoimperatorin	PTGS2
Isotanshinone II	NOS2
Isotanshinone II	DRD1
Isotanshinone II	CHRM3
Isotanshinone II	F2
Isotanshinone II	CHRM1
Isotanshinone II	ESR1
Isotanshinone II	AR
Isotanshinone II	SCN5A
Isotanshinone II	CHRM5
Isotanshinone II	PTGS2
Isotanshinone II	RXRA
Isotanshinone II	OPRD1
Isotanshinone II	ACHE
Isotanshinone II	ADRA1A
Isotanshinone II	CHRM2
Isotanshinone II	ADRB2
Isotanshinone II	OPRM1
Isotanshinone II	ESR2
Isotanshinone II	GABRA1
Isotanshinone II	DPP4
Isotanshinone II	GSK3B
Isotanshinone II	CDK2
Isotanshinone II	CHRNA7
Isotanshinone II	CHEK1
Isotanshinone II	PIM1
Isotanshinone II	CCNA2
Kaempferol	NOS2
Kaempferol	PTGS1
Kaempferol	AR
Kaempferol	PPARG
Kaempferol	PTGS2
Kaempferol	HSP90AA1
Kaempferol	PIK3CG
Kaempferol	NCOA2
Kaempferol	DPP4
Kaempferol	PRSS1
Kaempferol	PGR
Kaempferol	F2
Kaempferol	CHRM1
Kaempferol	NOS3
Kaempferol	GABRA2
Kaempferol	ACHE
Kaempferol	SLC6A2
Kaempferol	CHRM2
Kaempferol	ADRA1B
Kaempferol	GABRA1
Kaempferol	TOP2A
Kaempferol	F7

Kaempferol	RELA
Kaempferol	IKBKB
Kaempferol	AKT1
Kaempferol	BCL2
Kaempferol	BAX
Kaempferol	TNF
Kaempferol	JUN
Kaempferol	AHSA1
Kaempferol	CASP3
Kaempferol	MAPK8
Kaempferol	XDH
Kaempferol	MMP1
Kaempferol	STAT1
Kaempferol	CDK1
Kaempferol	HMOX1
Kaempferol	CYP3A4
Kaempferol	CYP1A2
Kaempferol	CYP1A1
Kaempferol	ICAM1
Kaempferol	SELE
Kaempferol	VCAM1
Kaempferol	NR1I2
Kaempferol	CYP1B1
Kaempferol	ALOX5
Kaempferol	HAS2
Kaempferol	GSTP1
Kaempferol	AHR
Kaempferol	PSMD3
Kaempferol	SLC2A4
Kaempferol	NR1I3
Kaempferol	INSR
Kaempferol	DIO1
Kaempferol	PPP3CA
Kaempferol	GSTM1
Kaempferol	GSTM2
Kaempferol	AKR1C3
Kaempferol	SLPI
Kaempferol	CALM1
Kaempferol	CALM2
Kaempferol	CALM3
Ligustilide	PTGS2
Ligustilide	GABRA1
Liquiritigenin	PTGS1
Liquiritigenin	ESR1
Liquiritigenin	PTGS2
Liquiritigenin	RXRA
Liquiritigenin	ADRB2
Liquiritigenin	PIK3CG
Liquiritigenin	MAOB
Liquiritigenin	SLC6A4
Liquiritigenin	PKIA
Luteolin	PTGS1
Luteolin	AR
Luteolin	PTGS2
Luteolin	HSP90AA1
Luteolin	PRSS1

Luteolin	NCOA2
Luteolin	DPP4
Luteolin	PIK3CG
Luteolin	RELA
Luteolin	EGFR
Luteolin	AKT1
Luteolin	VEGFA
Luteolin	CCND1
Luteolin	BCL2L1
Luteolin	CDKN1A
Luteolin	CASP9
Luteolin	MMP2
Luteolin	MMP9
Luteolin	MAPK1
Luteolin	IL10
Luteolin	RB1
Luteolin	CDK4
Luteolin	TNF
Luteolin	JUN
Luteolin	IL6
Luteolin	CASP3
Luteolin	TP53
Luteolin	NFKBIA
Luteolin	XDH
Luteolin	TOP1
Luteolin	MDM2
Luteolin	APP
Luteolin	MMP1
Luteolin	PCNA
Luteolin	ERBB2
Luteolin	PPARG
Luteolin	HMOX1
Luteolin	CASP7
Luteolin	ICAM1
Luteolin	MCL1
Luteolin	BIRC5
Luteolin	IL2
Luteolin	CCNB1
Luteolin	TYR
Luteolin	IFNG
Luteolin	IL4
Luteolin	TOP2A
Luteolin	GSTP1
Luteolin	XIAP
Luteolin	SLC2A4
Luteolin	INSR
Luteolin	CD40LG
Luteolin	NUF2
Luteolin	ADCY2
Luteolin	MET
Mairin	PGR
Mandenol	PTGS1
Mandenol	PTGS2
Mandenol	NCOA2
Manool	NCOA2
Methylenetanshinquinone	DRD1

Methylenetanshinquinone	CHRM3
Methylenetanshinquinone	F2
Methylenetanshinquinone	CHRM1
Methylenetanshinquinone	SCN5A
Methylenetanshinquinone	CHRM5
Methylenetanshinquinone	PTGS2
Methylenetanshinquinone	CA2
Methylenetanshinquinone	RXRA
Methylenetanshinquinone	OPRD1
Methylenetanshinquinone	ACHE
Methylenetanshinquinone	HTR2A
Methylenetanshinquinone	ADRA1A
Methylenetanshinquinone	CHRM2
Methylenetanshinquinone	ADRB2
Methylenetanshinquinone	SLC6A4
Methylenetanshinquinone	OPRM1
Methylenetanshinquinone	GABRA1
Methylenetanshinquinone	DPP4
Methylenetanshinquinone	CHRNA7
Methylenetanshinquinone	IGHG1
Methylenetanshinquinone	PRSS1
Methylenetanshinquinone	NCOA1
Miltionone I	PTGS1
Miltionone I	CHRM3
Miltionone I	CHRM1
Miltionone I	ESR1
Miltionone I	AR
Miltionone I	SCN5A
Miltionone I	F10
Miltionone I	PTGS2
Miltionone I	CA2
Miltionone I	RXRA
Miltionone I	OPRD1
Miltionone I	HTR2A
Miltionone I	ADRA1A
Miltionone I	CHRM2
Miltionone I	ADRA1B
Miltionone I	ADRB2
Miltionone I	TOP2A
Miltionone I	OPRM1
Miltionone I	NR3C1
Miltionone I	GSK3B
Miltionone I	CDK2
Miltionone I	CHRNA7
Miltionone I	IGHG1
Miltionone I	PIM1
Miltionone I	CCNA2
Miltionone I	NCOA2
Miltionone I	NCOA1
Miltionone II	F2
Miltionone II	PTGS2
Miltionone II	CA2
Miltionone II	ACHE
Miltionone II	PGR
Miltionone II	NR3C1
Miltionone II	NCOA2

Miltionone II	NCOA1
Miltipolone	ESR1
Miltipolone	ACHE
Miltirone	PTGS1
Miltirone	DRD1
Miltirone	CHRM3
Miltirone	CHRM1
Miltirone	ESR1
Miltirone	AR
Miltirone	DRD5
Miltirone	SCN5A
Miltirone	CHRM5
Miltirone	PTGS2
Miltirone	NOS3
Miltirone	CA2
Miltirone	ADRA2C
Miltirone	CHRM4
Miltirone	RXRA
Miltirone	OPRD1
Miltirone	ADRA1A
Miltirone	CHRM2
Miltirone	ADRA1B
Miltirone	SLC6A3
Miltirone	ADRB2
Miltirone	ADRA1D
Miltirone	TOP2A
Miltirone	OPRM1
Miltirone	CHRNA7
Miltirone	NCOA2
MOL005481	PTGS2
MOL007036	PTGS1
MOL007036	CHRM3
MOL007036	CHRM1
MOL007036	SCN5A
MOL007036	PTGS2
MOL007036	CA2
MOL007036	RXRA
MOL007036	ACHE
MOL007036	ADRA1A
MOL007036	ADRA1B
MOL007036	ADRB2
MOL007036	TOP2A
MOL007036	OPRM1
MOL007036	IGHG1
MOL007036	NCOA2
MOL007036	NCOA1
MOL007036	CALM1
MOL007036	CALM2
MOL007036	CALM3
MOL007048	PTGS2
MOL007050	NOS2
MOL007050	F2
MOL007050	ESR1
MOL007050	AR
MOL007050	PPARG
MOL007050	ESR2

MOL007050	MAPK14
MOL007050	GSK3B
MOL007050	CDK2
MOL007050	PIM1
MOL007050	CCNA2
MOL007070	F2
MOL007070	PTGS2
MOL007070	CA2
MOL007070	ACHE
MOL007070	DPP4
MOL007070	PRSS1
MOL007070	NCOA1
MOL007155	DRD1
MOL007155	CHRM3
MOL007155	F2
MOL007155	CHRM1
MOL007155	SCN5A
MOL007155	PTGS2
MOL007155	OPRD1
MOL007155	ACHE
MOL007155	ADRA1A
MOL007155	ADRB2
MOL007155	OPRM1
MOL007155	DPP4
MOL007155	CHRNA7
MOL007155	PRSS1
MOL007155	NCOA1
Myricanone	NOS2
Myricanone	PTGS1
Myricanone	F2
Myricanone	KCNH2
Myricanone	ESR1
Myricanone	AR
Myricanone	SCN5A
Myricanone	PPARG
Myricanone	PTGS2
Myricanone	F7
Myricanone	KDR
Myricanone	RXRA
Myricanone	PDE3A
Myricanone	ADRB2
Myricanone	ESR2
Myricanone	DPP4
Myricanone	MAPK14
Myricanone	GSK3B
Myricanone	CDK2
Myricanone	CHEK1
Myricanone	IGHG1
Myricanone	PIM1
Myricanone	CCNA2
Myricanone	NCOA1
Neocryptotanshinone	PTGS1
Neocryptotanshinone	CHRM3
Neocryptotanshinone	CHRM1
Neocryptotanshinone	SCN5A
Neocryptotanshinone	PPARG

Neocryptotanshinone	PTGS2
Neocryptotanshinone	CA2
Neocryptotanshinone	ADRA1B
Neocryptotanshinone	ADRB2
Neocryptotanshinone	ADRA1D
Neocryptotanshinone	TOP2A
Neocryptotanshinone	OPRM1
Neocryptotanshinone	CHRNA7
Neocryptotanshinone	IGHG1
Neocryptotanshinone	NCOA2
Neocryptotanshinone	NCOA1
Neocryptotanshinone II	PTGS1
Neocryptotanshinone II	DRD1
Neocryptotanshinone II	CHRM3
Neocryptotanshinone II	CHRM1
Neocryptotanshinone II	ESR1
Neocryptotanshinone II	AR
Neocryptotanshinone II	SCN5A
Neocryptotanshinone II	PTGS2
Neocryptotanshinone II	NOS3
Neocryptotanshinone II	CA2
Neocryptotanshinone II	CHRM4
Neocryptotanshinone II	RXRA
Neocryptotanshinone II	OPRD1
Neocryptotanshinone II	PDE3A
Neocryptotanshinone II	ADRA1A
Neocryptotanshinone II	CHRM2
Neocryptotanshinone II	ADRA1B
Neocryptotanshinone II	SLC6A3
Neocryptotanshinone II	ADRB2
Neocryptotanshinone II	ADRA1D
Neocryptotanshinone II	SLC6A4
Neocryptotanshinone II	OPRM1
Neocryptotanshinone II	GABRA1
Neocryptotanshinone II	GSK3B
Neocryptotanshinone II	CDK2
Neocryptotanshinone II	CHRNA7
Neocryptotanshinone II	PIM1
Neocryptotanshinone II	CCNA2
Paeoniflorgenone	GABRA1
Palbinone	PGR
Palbinone	NR3C2
Perlolyrine	F2
Perlolyrine	PTGS2
Perlolyrine	RXRA
Poriferast-5-en-3beta-ol	PGR
Poriferast-5-en-3beta-ol	NCOA2
Poriferasterol	PGR
Poriferasterol	NR3C2
Prolithospermic acid	NOS2
Prolithospermic acid	PTGS1
Prolithospermic acid	F2
Prolithospermic acid	ESR1
Prolithospermic acid	AR
Prolithospermic acid	PTGS2
Prolithospermic acid	PRSS1

Prolithospermic acid	CALM1
Prolithospermic acid	CALM2
Prolithospermic acid	CALM3
Przewalskin A	NR3C2
Przewalskin A	NR3C1
Przewalskin B	PTGS2
Przewalskin B	PGR
Przewalskin B	NR3C2
Przewalskin B	NR3C1
Przewalskin B	NCOA2
Przewalskin B	NCOA1
Przewaquinone B	F2
Przewaquinone B	PTGS2
Przewaquinone B	RXRA
Przewaquinone B	DPP4
Przewaquinone B	PIK3CG
Przewaquinone B	IGHG1
Przewaquinone B	PRSS1
Przewaquinone B	NCOA1
Przewaquinone C	PTGS1
Przewaquinone C	DRD1
Przewaquinone C	CHRM3
Przewaquinone C	F2
Przewaquinone C	CHRM1
Przewaquinone C	SCN5A
Przewaquinone C	CHRM5
Przewaquinone C	PTGS2
Przewaquinone C	CA2
Przewaquinone C	CHRM4
Przewaquinone C	OPRD1
Przewaquinone C	ACHE
Przewaquinone C	ADRA1A
Przewaquinone C	CHRM2
Przewaquinone C	ADRB2
Przewaquinone C	OPRM1
Przewaquinone C	GABRA1
Przewaquinone C	DPP4
Przewaquinone C	PIK3CG
Przewaquinone C	CHRNA7
Przewaquinone C	NCOA1
Przewaquinone E	F2
Przewaquinone E	PTGS2
Przewaquinone E	CA2
Przewaquinone E	ACHE
Przewaquinone E	DPP4
Przewaquinone E	NCOA1
Przewaquinone F	F2
Przewaquinone F	PTGS2
Przewaquinone F	DPP4
Przewaquinone F	PRSS1
Przewaquinone F	NCOA1
Quercetin	PTGS1
Quercetin	AR
Quercetin	PPARG
Quercetin	PTGS2
Quercetin	HSP90AA1

Quercetin	PIK3CG
Quercetin	NCOA2
Quercetin	DPP4
Quercetin	AKR1B1
Quercetin	PRSS1
Quercetin	TOP2A
Quercetin	F2
Quercetin	KCNH2
Quercetin	SCN5A
Quercetin	F10
Quercetin	ADRB2
Quercetin	MMP3
Quercetin	F7
Quercetin	NOS3
Quercetin	RXRA
Quercetin	ACHE
Quercetin	GABRA1
Quercetin	MAOB
Quercetin	RELA
Quercetin	EGFR
Quercetin	AKT1
Quercetin	VEGFA
Quercetin	CCND1
Quercetin	BCL2
Quercetin	BCL2L1
Quercetin	FOS
Quercetin	CDKN1A
Quercetin	EIF6
Quercetin	BAX
Quercetin	CASP9
Quercetin	PLAU
Quercetin	MMP2
Quercetin	MAPK1
Quercetin	IL10
Quercetin	EGF
Quercetin	RB1
Quercetin	TNF
Quercetin	JUN
Quercetin	IL6
Quercetin	AHSA1
Quercetin	CASP3
Quercetin	TP53
Quercetin	ELK1
Quercetin	NFKBIA
Quercetin	POR
Quercetin	ODC1
Quercetin	XDH
Quercetin	CASP8
Quercetin	TOP1
Quercetin	RAF1
Quercetin	SOD1
Quercetin	PRKCA
Quercetin	MMP1
Quercetin	HIF1A
Quercetin	STAT1
Quercetin	RUNX1T1

Quercetin	CDK1
Quercetin	HSPA5
Quercetin	ERBB2
Quercetin	ACACA
Quercetin	HMOX1
Quercetin	CYP3A4
Quercetin	CYP1A2
Quercetin	CAV1
Quercetin	MYC
Quercetin	F3
Quercetin	GJA1
Quercetin	CYP1A1
Quercetin	ICAM1
Quercetin	IL1B
Quercetin	CCL2
Quercetin	SELE
Quercetin	VCAM1
Quercetin	PTGER3
Quercetin	CXCL8
Quercetin	PRKCB
Quercetin	BIRC5
Quercetin	DUOX2
Quercetin	HSPB1
Quercetin	TGFB1
Quercetin	SULT1E1
Quercetin	MGAM
Quercetin	IL2
Quercetin	NR1I2
Quercetin	CYP1B1
Quercetin	CCNB1
Quercetin	PLAT
Quercetin	THBD
Quercetin	SERPINE1
Quercetin	COL1A1
Quercetin	IFNG
Quercetin	ALOX5
Quercetin	PTEN
Quercetin	IL1A
Quercetin	MPO
Quercetin	NCF1
Quercetin	ABCG2
Quercetin	HAS2
Quercetin	GSTP1
Quercetin	NFE2L2
Quercetin	NQO1
Quercetin	PARP1
Quercetin	AHR
Quercetin	PSMD3
Quercetin	SLC2A4
Quercetin	COL3A1
Quercetin	CXCL11
Quercetin	CXCL2
Quercetin	DCAF5
Quercetin	NR1I3
Quercetin	CHEK2
Quercetin	INSR

Quercetin	CLDN4
Quercetin	PPARA
Quercetin	PPARD
Quercetin	HSF1
Quercetin	CRP
Quercetin	CXCL10
Quercetin	CHUK
Quercetin	SPP1
Quercetin	RUNX2
Quercetin	RASSF1
Quercetin	E2F1
Quercetin	E2F2
Quercetin	ACPP
Quercetin	CTSD
Quercetin	IGFBP3
Quercetin	IGF2
Quercetin	CD40LG
Quercetin	IRF1
Quercetin	ERBB3
Quercetin	PON1
Quercetin	DIO1
Quercetin	PCOLCE
Quercetin	NPEPPS
Quercetin	HK2
Quercetin	NKX3-1
Quercetin	RASA1
Quercetin	GSTM1
Quercetin	GSTM2
Salvianolic acid G	PTGS2
Salvilenone	PTGS1
Salvilenone	ESR1
Salvilenone	AR
Salvilenone	CHRM5
Salvilenone	PTGS2
Salvilenone	HTR3A
Salvilenone	ESR2
Salvilenone	PIM1
Salvilenone I	PTGS2
Salvilenone I	RXRA
Salvilenone I	ACHE
Salvilenone I	PGR
Salvilenone I	NR3C1
Salvilenone I	NCOA2
Salvilenone I	NCOA1
Salviolone	PTGS1
Salviolone	DRD1
Salviolone	CHRM3
Salviolone	F2
Salviolone	CHRM1
Salviolone	DRD5
Salviolone	SCN5A
Salviolone	CHRM5
Salviolone	PTGS2
Salviolone	ADRA2A
Salviolone	HTR1A
Salviolone	HTR3A

Salviolone	GABRA2
Salviolone	CHRM4
Salviolone	OPRD1
Salviolone	ACHE
Salviolone	PDE3A
Salviolone	HTR2A
Salviolone	GABRA5
Salviolone	SLC6A2
Salviolone	ADRA1A
Salviolone	GABRA3
Salviolone	HTR2C
Salviolone	CHRM2
Salviolone	ADRA2B
Salviolone	ADRA1B
Salviolone	SLC6A3
Salviolone	ADRB2
Salviolone	CHRNA2
Salviolone	SLC6A4
Salviolone	DRD2
Salviolone	OPRM1
Salviolone	GABRA1
Salviolone	HTR1B
Salviolone	CHRNA7
Salviolone	GABRA6
Salviolone	GABRG3
Salviolone	GABRE
Sclareol	PTGS2
Senkyunolide I	PTGS1
Senkyunolide I	CHRM1
Senkyunolide I	PTGS2
Senkyunolide I	CHRM2
Senkyunolide I	ADRA2B
Senkyunolide I	ADRB2
Senkyunolide I	SLC6A4
Senkyunolide I	GABRA1
Senkyunolide I	MAOB
Senkyunolide I	PKIA
Sitosterol	PGR
Sitosterol	NCOA2
Sitosterol	NR3C2
Sitosterol alpha1	PGR
Sitosterol alpha1	PTGS2
Sitosterol alpha1	GABRA1
Sitosterol alpha1	ADH1C
Sitosterol alpha1	NR3C2
Stachyose	PTGS1
Stachyose	PTGS2
Stachyose	GABRA1
Stachyose	MAN2A1
Stigmasterol	PGR
Stigmasterol	NR3C2
Stigmasterol	NCOA2
Stigmasterol	ADH1C
Stigmasterol	IGHG1
Stigmasterol	RXRA
Stigmasterol	NCOA1

Stigmasterol	PTGS1
Stigmasterol	PTGS2
Stigmasterol	ADRA2A
Stigmasterol	SLC6A2
Stigmasterol	SLC6A3
Stigmasterol	ADRB2
Stigmasterol	AKR1B1
Stigmasterol	PLAU
Stigmasterol	LTA4H
Stigmasterol	MAOB
Stigmasterol	MAOA
Stigmasterol	CTRB1
Stigmasterol	CHRM3
Stigmasterol	CHRM1
Stigmasterol	ADRB1
Stigmasterol	SCN5A
Stigmasterol	HTR2A
Stigmasterol	ADRA1A
Stigmasterol	GABRA3
Stigmasterol	CHRM2
Stigmasterol	ADRA1B
Stigmasterol	GABRA1
Stigmasterol	CHRNA7
Sugiol	CHRM3
Sugiol	CHRM1
Sugiol	SCN5A
Sugiol	CHRM5
Sugiol	PTGS2
Sugiol	CA2
Sugiol	CHRM4
Sugiol	OPRD1
Sugiol	ACHE
Sugiol	ADRA1A
Sugiol	CHRM2
Sugiol	ADRA1B
Sugiol	ADRB2
Sugiol	ADRA1D
Sugiol	DRD2
Sugiol	OPRM1
Sugiol	CHRNA7
Tanshinaldehyde	DRD1
Tanshinaldehyde	F2
Tanshinaldehyde	CHRM1
Tanshinaldehyde	PTGS2
Tanshinaldehyde	OPRD1
Tanshinaldehyde	ACHE
Tanshinaldehyde	HTR2A
Tanshinaldehyde	ADRB2
Tanshinaldehyde	OPRM1
Tanshinaldehyde	DPP4
Tanshinaldehyde	CHRNA7
Tanshinaldehyde	PRSS1
Tanshinaldehyde	NCOA1
Tanshindiol B	F2
Tanshindiol B	PTGS2
Tanshindiol B	CA2

Tanshindiol B	ACHE
Tanshindiol B	DPP4
Tanshindiol B	NCOA1
Tanshinone IIA	CHRM5
Tanshinone IIA	PTGS2
Tanshinone IIA	CHRM4
Tanshinone IIA	OPRD1
Tanshinone IIA	ACHE
Tanshinone IIA	ADRA1A
Tanshinone IIA	CHRM2
Tanshinone IIA	ADRB2
Tanshinone IIA	OPRM1
Tanshinone IIA	DPP4
Tanshinone IIA	CHRNA7
Tanshinone IIA	NCOA1
Tanshinone IIA	RXRA
Tanshinone IIA	RELA
Tanshinone IIA	BCL2
Tanshinone IIA	FOS
Tanshinone IIA	CDKN1A
Tanshinone IIA	MMP9
Tanshinone IIA	JUN
Tanshinone IIA	AHSA1
Tanshinone IIA	CASP3
Tanshinone IIA	TP53
Tanshinone IIA	NFKBIA
Tanshinone IIA	FASN
Tanshinone IIA	EDN1
Tanshinone IIA	CYP3A4
Tanshinone IIA	CYP1A2
Tanshinone IIA	MYC
Tanshinone IIA	CYP1A1
Tanshinone IIA	NR1I2
Tanshinone IIA	NPM1
Tanshinone IIA	ECE1
Tanshinone IIA	PARP4
Tanshinone IIA	CALCR
Tanshinone IIA	ITGB3
Tanshinone VI	PTGS1
Tanshinone VI	ESR1
Tanshinone VI	AR
Tanshinone VI	SCN5A
Tanshinone VI	PPARG
Tanshinone VI	F10
Tanshinone VI	PTGS2
Tanshinone VI	IGHG1
Tanshinone VI	NCOA2
Tanshinone VI	NCOA1
Tanshinone VI	CALM1
Tanshinone VI	CALM2
Tanshinone VI	CALM3
Telocinobufagin	NR3C2
Telocinobufagin	NR3C1
Telocinobufagin	NOS2
Telocinobufagin	PTGS1
Telocinobufagin	DRD1

Telocinobufagin	CHRM3
Telocinobufagin	F2
Telocinobufagin	KCNH2
Telocinobufagin	CHRM1
Telocinobufagin	AR
Telocinobufagin	SCN5A
Telocinobufagin	PPARG
Telocinobufagin	F10
Telocinobufagin	CHRM5
Tetrahydroalstonine	PTGS2
Tetrahydroalstonine	ADRA2C
Tetrahydroalstonine	CHRM4
Tetrahydroalstonine	OPRD1
Tetrahydroalstonine	ACHE
Tetrahydroalstonine	HTR2A
Tetrahydroalstonine	ADRA1B
Tetrahydroalstonine	ADRB2
Tetrahydroalstonine	ADRA1D
Tetrahydroalstonine	SLC6A4
Tetrahydroalstonine	OPRM1
Tetrahydroalstonine	DPP4
Tetrahydroalstonine	PRSS1
Tetrahydroalstonine	CALM1
Tetrahydroalstonine	CALM2
Tetrahydroalstonine	CALM3
Wallichilide	PTGS2
Wallichilide	NR3C2
Wallichilide	NR3C1
Wallichilide	NCOA2
