# Supplementary material for: Systematic Pharmacology-Based Strategy to Explore the Molecular Network Mechanism of Modified Taohong Siwu Decoction in the Treatment of Premature Ovarian Failure
Source: Evid Based Complement Alternat Med. 2022 Jan 21;2022:3044463. doi: 10.1155/2022/3044463 (PMC8799328; doi:10.1155/2022/3044463)
Supplement: Supplementary Materials — Figure S1: the results of HPLC. Table S1: components and targets of MTHSWD. Table S2: POF genes. Table S3: enrichment analysis of MTHSWD-POF PPI network. [file 3044463.f1.zip › 3044463.f1/Table S2.pdf]

Table S2 POF genes
POF1B
STAG3
NOBOX
FMR1
FOXL2
BNC1
FIGLA
FRAXA
GALT
MCM9
PMM2
RAD54L
CHEK2
JAK2
BRDT
INSL6
SGO2
BMP15
ERCC6
LOC107032825
DIAPH2
MCM8
GDF9
HFM1
NR5A1
MSH5
FANCM
SYCE1
PGBD3
BRCA2
XRCC2
C14orf39
HSF2BP
MSH5-SAPCD1
ABCD1
CYP19A1
CYP17A1
AFF2
IGF2R
INHA
KDR
FANCA
LRRC41
BMP6
ADAMTS1
AFP
KHDRBS1
BRWD1
ADAMTS6
DPPA2
ATG9B
DMRT3
NBN
EIF2B2
MSH4

LARS2
ZNF711
POLG
AARS2
TP63
ERCC1
PEX6
PREPL
LMNA
FSHR
POLR1C
MRPS22
TWNK
TG
IGSF10
DUPXQ27.3Q28
DMC1
DACH2
RYR3
PRLR
ESR1
XPNPEP2
CLPP
POLG2
NOS3
WT1
FOXP2
AR
NR5A2
ATM
FOXE1
SHBG
AMH
PCMT1
LARS2-AS1
ERAL1
OSR2
FOXO3
NOTCH2
POR
NTRK1
MECOM
SIRT6
THBS1
RICTOR
RAD51C
MLH3
SH2B1
ZNF462
REC8
MIR4713HG
PIRC66
IGF1
PRL
PGRMC1
ADAMTS19

CYP21A2
MYC
PRKCD
FAS
CASP10
FASLG
RASGRP1
PTEN
EIF2B4
LHX8
GPR3
ANAPC1
MCM3AP
GNRH1
NANOS3
INS
CYP11A1
LHCGR
ESR2
SPO11
WNT4
AMHR2
SOHLH2
NR0B1
ZIC1
HNF1B
SOX14
FOXE3
ZFHX4
ZIC4
BPESC1
PISRT1
SPATA8-AS1
DAZL
HSD17B4
FST
RECQL4
MILR1
KITLG
FSHB
PNPLA7
POU5F1
SOHLH1
PABPC5
AIRE
PSMC3IP
GALK1
MTHFR
USP9X
LEPR
TPO
IGFBP1
PRKD1
C3
GLI1
FANCC

FYN
CHD7
ADAMTS5
ARNTL
ATG7
PRIM1
ACKR3
PARD3
BRD3
MOV10
PADI6
ATG2A
WHAMM
AOPEP
OR8J1
CDKN1B
IGF2
NLRP5
BRCA1
SHOX
PGR
STAR
GJA4
GNRHR
TGFBR3
AKT1
POMC
KISS1R
HSD3B1
KISS1
EIF2B5
ACSL6
MTOR
TP53
SOX9
DDX4
CPEB1
EIF4ENIF1
CXCL12
BBS9
UGT1A1
CASP3
ALB
PTH
TLK1
DMRT1
BRSK1
SYCP3
ZP3
ZP2
YBX2
SMC1B
MND1
MAMLD1
STRA8
FNDC4

EPPIN
NLRP11
NUDT10
MEIOB
NXF5
DPPA3
RFPL4A
CFAP47
TMEM150B
MIR146A
INHBB
BDNF
NUP107
ZMPSTE24
UGT1A6
SYNE2
KIAA0319L
UGT1A8
CRYZL1
TAAR8
SALL4
TAF4B
GH1
IGFBP3
BLM
LEP
INHBA
GHR
RPL10
SRY
KIT
NTRK3
ACVR2B
IL2RB
PCSK1
NOG
ADIPOQ
AGTR2
CGA
HSD3BP4
LGR6
TSHB
HELQ
NXF2
MIR23A
MIR22
COL1A1
XIAP
FOXO1
VEGFA
TYMS
SKP2
EXO1
BCKDHB
CITED2
FOXO4

LAMC1
MTRR
NCOA6
ADAMTS16
H19
MIR196A2
RPS20P23
LEPQTL1
ADIPQTL4
KDM6A
PTPN22
HOXD13
PAPPA
SHOX2
RPS4X
SERPINA7
EFHC2
ARSL
RPS4Y1
TSPY1
CGB3
LINC01436
MIR663AHG
RB1
E2F1
H2AC18
IGF1R
KRAS
RAD50
TNF
SIRT1
WRN
MRE11
EIF2B3
STAT3
MT-CO1
USP8
B4GALNT1
INVS
NPHP1
EIF2B1
IQCB1
CDH23
RIN2
SDCCAG8
CEP164
CEP290
NPHP4
RCBTB1
NPHP3
TRAF3IP1
POLR3H
THOC6
WDR19
DCAF17
TTI2

SPIDR
ATR
JAK1
RAD51
MDM2
CASP8
HRAS
TERT
ALPL
IL2RA
PTPRC
CD4
CD19
NRAS
RIPK1
TNFRSF1A
ACTA2
LIG4
TNFAIP3
ARF1
IL10
FANCD2
HDAC9
FOXP3
FADD
LIG1
SH2D1A
ALPP
CD28
CFLAR
IL7R
CD8A
MATK
RAG1
PML
RPA1
TNFSF10
SPP1
SMC1A
XRCC6
BCL2L11
AICDA
CCR6
CD27
IL2
CTLA4
DCLRE1C
KPNA2
PTPN3
PRF1
XRCC5
ALPI
UNC13D
ARFGAP1
FAF1
RAD17

RIPK3
COL5A2
FANCF
NHEJ1
MLKL
SLC30A5
TRIP11
TERF1
ARFGAP3
NUP85
LRBA
NUP133
MDC1
TERF2
TCN1
FANCB
CD69
NUP160
MAGT1
ICOSLG
PIR
RAB3IL1
ANKRD49
AHCTF1
H2AX
ARFGAP2
MT-CYB
ALPG
OSGIN2
MIR21
MT-TK
MT-TF
MT-TH
TH2LCRR
SLC25A4
RRM2B
SUCLA2
TYMP
SUCLG1
DGUOK
RAN
PURA
PDGFRL
MPV17
IMMT
MSTO1
SSBP1
H1-1
MCM3AP-AS1
FMR1-AS1
CYP2B6
CYP2C9
CYP2C19
CYP3A5
UBE2I
LIF

PAEP
LHB
ADRB2
ACTB
SMAD3
ACTN4
HSPA5
PIAS1
SMAD5
ZFX
LOC108863620
FGFR1
INSR
PTPN11
TGFBR1
DDC
NFKB1
NFKB2
PPARG
TH
ACVR1
COMT
CYP3A4
NEK2
MAPK14
TGFB1
VDR
DBH
DRD2
BMPR1A
ACE
BMPR1B
BMPR2
APOE
NR3C1
MAOA
SLC6A3
SERPINE1
ROCK1
TGFB2
SMAD4
PCSK9
TGFBR2
CYP2A6
CYP1B1
BMP4
ACVRL1
AGT
CHRNA4
IL6
GABBR2
OPRM1
LDLR
DRD5
F2
HTR2C

HSD17B3
GABRB2
HTR2A
IL1RN
MC2R
SSTR2
SLC6A4
CYP1A1
BUB3
ACVR1B
AKR1C2
CHRNA2
CNR1
CCR3
HTR1A
CD40LG
GNB3
FMO3
LIPE
SMAD2
SMAD6
CYP1A2
BCAT1
BMP2
ACVR2A
ABCC8
ADAMTS13
AKR1C3
CHRNA1
CHRNA7
F5
HSD3B2
HSP90AB1
OPRK1
NPY
NKX2-1
MC4R
SUMO1
SCARB1
UCP2
TWIST1
SSTR3
TGFB3
DRD3
DOT1L
DRD1
BMP7
ALDH1B1
CHRNA5
CHRNB2
CHRNB1
ARRB2
CRHR1
HSD17B2
FTO
OPRD1

IGFALS
SLC12A7
UCP1
TPH1
UGT2B7
SRD5A1
CHRNA3
CHRNB4
GHRHR
NR0B2
PDYN
PROK2
PPIF
SSTR1
SMAD1
CBR3
CHRNE
CHRNA9
CRHR2
CCL5
HTR5A
IGFBP2
HSD17B1
GHRL
HLA-DQB1
FMO1
INSRR
MAOB
MC3R
LEFTY2
POU1F1
SULT1A1
SSTR4
SREBF2
SRD5A2
RETN
SMAD7
NODAL
UCP3
BMP3
ACVR1C
CHRNA6
CHRND
CHRNB3
IGFBP6
GDF2
DDX20
GDF6
CCK
IGFBP4
IGFBP5
LEFTY1
PROKR2
SLC2A6
UGT2B4
SST

SSTR5
PCM1
ECT2
BMP5
CHRNG
CHFR
B3GNT5
GDF3
EME1
GDF11
MCHR1
TAX1BP1
TACC1
SLC2A12
SIM1
NEK4
DOK5
EML1
FMN2
SPAST
BMP10
BMP8B
CHRNA10
GHRH
HBE1
ADAMTS9
ANKK1
INHBE
GDF7
PENK
EBF2
ANOS1
CEP70
FGFR1OP2
FOXF2
INHBC
HIVEP1
GDF1
GDF10
LEPROTL1
TM7SF3
ZNF71
MFHAS1
CGB5
CITED4
LOC108783649
AKT2
AGTR1
GJA1
IRAK1
PTH1R
TSC2
NFE2L2
CARD11
MCL1
TRAF6

SOX2
CRP
LATS1
OXTR
CRH
MSTN
PLIN1
HTT
MLX
SELENBP1
CBX2
CALCA
FBL
NOP56
NRF1
BGLAP
CDK12
OXT
SHANK3
BLZF1
GRK3
NANOG
MOS
KARS1
DCAF13
ATP5PF
TENT5A
DAZ4
DNAI7
XIST
MIR27A
LOC100287033
LOC108964933
LOC110386951
LOC110408762
PIK3CA
FLJ22792
MER3
POF6
BPES
POF14
POF13
POF8
POF5
FANCU
FTZF1
CKN2
POF12
KIAA1596
SIX6OS1
POF16
POF10
POF19
GDF9B
DIA
POF9

BPES1
SPGF50
FTZ1
COFS1
SPGF15
SPGF28
SPGF52
ODG2
POF2B
POF2A
POF1
PFRK
POF17
SF1
CSB
POF15
POF18
POF4
POF3
AD4BP
ARMD5
POF7
UVSS1
SRXY3
POF11
SPGF8
SRXX4
