# Supplementary material for: Systematic Pharmacology-Based Strategy to Explore the Molecular Network Mechanism of Modified Taohong Siwu Decoction in the Treatment of Premature Ovarian Failure
Source: Evid Based Complement Alternat Med. 2022 Jan 21;2022:3044463. doi: 10.1155/2022/3044463 (PMC8799328; doi:10.1155/2022/3044463)
Supplement: Supplementary Materials — Figure S1: the results of HPLC. Table S1: components and targets of MTHSWD. Table S2: POF genes. Table S3: enrichment analysis of MTHSWD-POF PPI network. [file 3044463.f1.zip › 3044463.f1/Table S3.pdf]

Table S3 Enrichmen

Category	Term
	GO:0010862
	GO:0045944
	GO:0042493
	GO:0060395
	GO:0045893
	GO:0007568
	GO:0045471
	GO:0010628
	GO:0001666
	GO:0008284
	GO:0007271
	GO:0030509
	GO:0043066
	GO:0001541
	GO:0042981
Biological	GO:0007179
	GO:0007165
	GO:0043408
	GO:0008584
	GO:0008285
	GO:0032355
	GO:0035094
	GO:0032924
	GO:0043627
	GO:0048468
	GO:0040007
	GO:0071456
	GO:0007267
	GO:0010629
	GO:0032496
	GO:0005615
	GO:0005829
	GO:0005576
	GO:0005892
	GO:0043235
	GO:0005654
	GO:0005887
	GO:0045211
	GO:0045121
	GO:0009897
	GO:0005667
	GO:0009986
	GO:0005886
	GO:0043005
	GO:0005737
Cell Compo	GO:0005634
	GO:0005901
	GO:0043025
	GO:0043234
	GO:0031090
	GO:0030425
	GO:0016605
	GO:0000784

GO:000078
GO:003109
GO:000080
GO:004367
GO:003126
GO:004847
GO:003126

GO:000516
GO:000808
GO:001989
GO:000512
GO:000551
GO:004280
GO:000814
GO:004216
GO:000488
GO:000517
GO:004280
GO:001546
GO:001527
GO:004698
GO:000368
Molecular f GO:000813
GO:000523
GO:000467
GO:004356
GO:001990
GO:000552
GO:000549
GO:000510
GO:003162
GO:003023
GO:003199
GO:000370
GO:000487
GO:002003
GO:003199

hsa04080
hsa04350
hsa04068
hsa04913
hsa04668
hsa04917
hsa04210
hsa04151
hsa04660
hsa00140
hsa04066
hsa04060
hsa04722
hsa04115
hsa04064
hsa04621
hsa04915

	hsa04620
Signaling P	hsa04010
	hsa04912
	hsa04370
	hsa04914
	hsa04662
	hsa04390
	hsa04014
	hsa04020
	hsa04931
	hsa04062
	hsa04630
	hsa04910
	hsa04664
	hsa04152
	hsa04150
	hsa05320
	hsa04650
	hsa04750
	hsa04672

ıt Analysis of MTHSWD-POF PPI Network

Description
positive regulation of pathway-restricted SMAD protein phosphorylation
positive regulation of transcription from RNA polymerase II promoter
response to drug
SMAD protein signal transduction
positive regulation of transcription, DNA-templated
aging
response to ethanol
positive regulation of gene expression
response to hypoxia
positive regulation of cell proliferation
synaptic transmission, cholinergic
BMP signaling pathway
negative regulation of apoptotic process
ovarian follicle development
regulation of apoptotic process
transforming growth factor beta receptor signaling pathway
signal transduction
regulation of MAPK cascade
male gonad development
negative regulation of cell proliferation
response to estradiol
response to nicotine
activin receptor signaling pathway
response to estrogen
cell development
growth
cellular response to hypoxia
cell-cell signaling
negative regulation of gene expression
response to lipopolysaccharide
extracellular space
cytosol
extracellular region
acetylcholine-gated channel complex
receptor complex
nucleoplasm
integral component of plasma membrane
postsynaptic membrane
membrane raft
external side of plasma membrane
transcription factor complex
cell surface
plasma membrane
neuron projection
cytoplasm
nucleus
caveola
neuronal cell body
protein complex
organelle membrane
dendrite
PML body
nuclear chromosome, telomeric region

chromosome, telomeric region
platelet alpha granule lumen
lateral element
axon terminus
CD95 death-inducing signaling complex
perinuclear region of cytoplasm
death-inducing signaling complex

transforming growth factor beta receptor binding
growth factor activity
enzyme binding
cytokine activity
protein binding
protein homodimerization activity
drug binding
acetylcholine binding
acetylcholine-activated cation-selective channel activity
hormone activity
identical protein binding
acetylcholine receptor activity
ligand-gated ion channel activity
protein heterodimerization activity
chromatin binding
transcription factor binding
extracellular ligand-gated ion channel activity
transmembrane receptor protein serine/threonine kinase activity
sequence-specific DNA binding
protein kinase binding
ATP binding
steroid binding
receptor binding
ubiquitin protein ligase binding
nitric-oxide synthase regulator activity
insulin-like growth factor II binding
steroid hormone receptor activity
RNA polymerase II transcription factor activity, ligand-activated sequence-specific DNA binding
heme binding
insulin-like growth factor I binding

Neuroactive ligand-receptor interaction
TGF-beta signaling pathway
FoxO signaling pathway
Ovarian steroidogenesis
TNF signaling pathway
Prolactin signaling pathway
Apoptosis
PI3K-Akt signaling pathway
T cell receptor signaling pathway
Steroid hormone biosynthesis
HIF-1 signaling pathway
Cytokine-cytokine receptor interaction
Neurotrophin signaling pathway
p53 signaling pathway
NF-kappa B signaling pathway
NOD-like receptor signaling pathway
Estrogen signaling pathway

Toll-like receptor signaling pathway
MAPK signaling pathway
GnRH signaling pathway
VEGF signaling pathway
Progesterone-mediated oocyte maturation
B cell receptor signaling pathway
Hippo signaling pathway
Ras signaling pathway
Calcium signaling pathway
Insulin resistance
Chemokine signaling pathway
Jak-STAT signaling pathway
Insulin signaling pathway
Fc epsilon RI signaling pathway
AMPK signaling pathway
mTOR signaling pathway
Autoimmune thyroid disease
Natural killer cell mediated cytotoxicity
Inflammatory mediator regulation of TRP channels
Intestinal immune network for IgA production

Count	%	PValue
37	4.506699	6.51E-38
144	17.53959	1.68E-34
75	9.135201	3.24E-32
37	4.506699	1.01E-31
97	11.81486	1.65E-31
49	5.968331	6.47E-25
40	4.872107	6.98E-25
61	7.429963	8.63E-25
48	5.846529	3.88E-23
78	9.500609	5.30E-22
24	2.923264	1.11E-21
32	3.897686	1.77E-21
76	9.257004	2.17E-21
25	3.045067	2.48E-21
50	6.090134	1.51E-20
33	4.019488	1.20E-19
129	15.71255	1.34E-19
23	2.801462	7.45E-19
32	3.897686	2.62E-18
65	7.917174	5.79E-18
31	3.775883	9.32E-18
21	2.557856	2.42E-17
15	1.82704	3.43E-17
26	3.16687	7.45E-17
21	2.557856	1.82E-16
18	2.192448	4.18E-16
30	3.65408	5.71E-16
47	5.724726	4.87E-15
34	4.141291	7.99E-15
37	4.506699	1.06E-14
159	19.36663	8.94E-31
247	30.08526	2.98E-18
148	18.0268	6.38E-18
17	2.070646	1.06E-16
32	3.897686	4.48E-15
208	25.33496	6.88E-15
125	15.22533	1.27E-13
39	4.750305	1.42E-13
34	4.141291	1.50E-10
34	4.141291	3.69E-10
32	3.897686	5.06E-10
57	6.942753	3.95E-09
254	30.93788	5.09E-09
34	4.141291	5.96E-09
307	37.39342	7.25E-09
316	38.48965	8.09E-09
17	2.070646	1.71E-08
39	4.750305	2.39E-08
46	5.602923	2.66E-08
19	2.314251	4.14E-08
40	4.872107	4.15E-08
20	2.436054	5.30E-08
22	2.679659	2.84E-07

13	1.583435	5.35E-07
14	1.705238	6.25E-07
8	0.974421	8.79E-07
13	1.583435	8.83E-07
6	0.730816	9.97E-07
55	6.699147	2.04E-06
6	0.730816	3.36E-06
32	3.897686	1.45E-32
48	5.846529	1.49E-24
68	8.282582	2.18E-24
47	5.724726	5.62E-22
545	66.38246	8.74E-21
96	11.69306	1.12E-19
30	3.65408	2.43E-19
19	2.314251	1.38E-18
17	2.070646	1.04E-16
30	3.65408	1.88E-16
91	11.08404	2.88E-16
16	1.948843	4.37E-15
19	2.314251	5.20E-15
65	7.917174	1.39E-14
57	6.942753	1.24E-13
46	5.602923	9.67E-13
16	1.948843	5.91E-12
9	1.096224	2.19E-10
60	7.308161	4.11E-10
49	5.968331	4.29E-10
124	15.10353	8.20E-10
13	1.583435	1.11E-09
46	5.602923	1.60E-09
40	4.872107	3.25E-09
8	0.974421	4.12E-09
8	0.974421	4.12E-09
17	2.070646	4.13E-09
14	1.705238	4.84E-09
26	3.16687	6.10E-09
9	1.096224	1.06E-08
89	10.84044	2.91E-30
45	5.481121	7.89E-26
50	6.090134	4.58E-20
30	3.65408	2.14E-19
41	4.99391	5.62E-17
32	3.897686	1.28E-15
29	3.532278	1.11E-14
**73**	8.891596	1.41E-13
35	4.263094	3.79E-13
25	3.045067	9.40E-12
32	3.897686	1.98E-11
53	6.455542	1.71E-10
34	4.141291	5.36E-10
24	2.923264	2.12E-09
27	3.288672	5.54E-09
20	2.436054	6.74E-08
27	3.288672	1.05E-07

28	3.410475	1.19E-07
48	5.846529	1.48E-07
25	3.045067	3.00E-07
20	2.436054	3.14E-07
24	2.923264	5.06E-07
21	2.557856	5.68E-07
33	4.019488	7.91E-07
42	5.115713	1.80E-06
35	4.263094	4.94E-06
25	3.045067	8.13E-06
35	4.263094	1.18E-05
29	3.532278	2.48E-05
27	3.288672	7.43E-05
17	2.070646	1.28E-04
24	2.923264	2.15E-04
15	1.82704	2.52E-04
14	1.705238	2.87E-04
22	2.679659	0.001238
19	2.314251	0.001266
10	1.218027	0.015452

Genes
ACVRL1, BMP10, BMPR2, MSTN, ACVR1B, BMP15, LEFTY2, ACVR1, GDF10, GDF11, TGFB2, LEFTY
RB1, APP, SERPINE1, AHR, NR3C1, ELK1, TNF, SOX2, MYC, SALL4, AKT1, SOX9, TP63, ACVR1, OSR
CDKN1A, OXTR, CDKN1B, PTEN, HTR2C, HTR2A, COMT, SLC6A2, ADRA1A, SLC6A3, SLC6A4, ICAI
BMP10, MSTN, AFP, BMP15, LEFTY2, GDF10, SMAD2, GDF11, SMAD1, TGFB2, LEFTY1, JUN, SMAD
RB1, AHR, ELK1, TNF, SOX2, MYC, CHEK2, SOX9, TP63, ACVR1, OSR2, ANKRD49, HNF1B, FOXP3, I
PTEN, HTR2A, PTH1R, MPO, ADRA1A, SLC6A3, CASP9, CASP7, AKT1, AMH, POLG, IL10, IGFBP1, IG
MAOB, MSTN, PTH, GSTP1, PTEN, SLC2A4, TYMS, SLC6A3, ICAM1, RPS4X, CASP8, CCND1, CNR1, I
CITED2, PTPN22, ARRB2, BRCA1, TNF, SLC6A4, GJA1, MYC, AMH, HRAS, NKX3-1, KDM6A, OSR2, I
ACVRL1, CDKN1B, CITED2, CHRNA4, CHRNA7, PLAT, THBS1, HIF1A, SLC6A4, DPP4, PLAU, CASP3
CDKN1B, CHRM1, BNC1, PTEN, ADRA1D, FASLG, HTR2A, PTH1R, GLI1, IGF1R, RPS4X, MYC, KDR,
CHRM2, CHRNA1, CHRNB2, NQO1, CHRM3, CHRNB1, CHRNA3, CHRNB4, CHRM1, CHRNA2, CHRNI
ACVRL1, BMP10, BMPR2, BMP15, ACVR1, GDF10, SMAD1, SMAD4, USP9X, NOG, GDF2, BMP8B, GD
GSK3B, CDKN1A, FOXE3, CDKN1B, CITED2, PTEN, HSPB1, FMN2, MPO, IGF1R, IKBKB, INSL6, MYC
SPO11, ICAM1, BMP15, STRA8, SOHLH1, MYC, EIF2B5, EIF2B4, SMAD4, EIF2B2, FOXL2, INHBB, INH
BMP10, MSTN, BRCA1, BMP15, CASP9, CASP8, CASP10, SOX9, JAK2, SKP2, TP63, LEFTY1, IGFBP3,
ACVRL1, AMHR2, MSTN, CITED2, ARRB2, CCL2, LEFTY2, ACVR1, GDF10, SMAD2, SMAD1, TGFB2,
CHRM3, CHRM1, AMHR2, CHRM4, NR3C1, IGF1R, NR3C2, GJA1, PLAU, SALL4, AKT2, TNFSF10, AK
GDF10, GDF11, BMP10, LEFTY1, MSTN, TGFB3, GDF2, GDF1, BMP8B, INHBB, GDF3, INHBA, GDF6,
AMHR2, CITED2, LHB, INSL6, BCL2L11, TNFSF10, SOX9, NKX3-1, WNT4, NCOA1, TGFB2, LHCGR, S
RB1, ACVRL1, CDKN1A, CDKN1B, CXCL8, PTEN, PTH1R, ADRA1A, ADAMTS1, CYP1B1, JAK2, HRA
OXTR, CDKN1B, PCNA, GSTP1, PTEN, OXT, PTGS2, SLC6A4, GHR, CASP9, CASP8, CCND1, MYC, CA
CHRNA1, CHRNB2, EDN1, CHRNB4, CHRNA2, VCAM1, CHRNB3, CHRNA4, CHRNA7, CHRNA6, SLC
SMAD2, ACVR1, ACVRL1, BMP10, BMPR2, SMAD3, GDF2, INHBB, INHBA, GDF6, ACVR1B, ACVR2E
MSTN, CITED2, CITED4, BRCA1, GHRHR, CCND1, BGLAP, CA2, HMOX1, MAPK1, TSHB, HSP90AA1,
GDF10, GDF11, LEFTY1, FOXE3, MSTN, GDF2, GDF1, BMP8B, INHBB, GDF3, INHBA, GDF6, INHBC,
GDF11, GDF2, BMP8B, INHBB, GDF3, INHBA, GDF6, BMP7, FOXP2, MTOR, BMP6, BMP5, GDF7, VEG
OPRD1, PTEN, TWIST1, FMN2, SLC2A4, FOXO3, PTGS2, HIF1A, ICAM1, NPEPPS, CCNB1, IRAK1, TEI
PCSK1, CALCA, PTH, LHB, ADRA1D, ADRB1, FASLG, ADRB2, ADRA1B, ADRA1A, INS, GJA1, CCL5,
ACVRL1, RB1, OPRD1, CDKN1A, CITED2, PTPN22, ACVR1B, TNF, GJA1, CCNB1, TERT, AKT1, FYN,
MAOB, PTPN22, FASLG, COMT, PTGS2, MPO, CXCL2, CASP9, THBD, CASP8, IRAK1, CNR1, CASP3, I

APP, IL1RN, SERPINE1, PLAT, MPO, TNF, ACTB, ICAM1, PLAU, CHEK1, TNFSF10, EPPIN, IGFBP1, IC
APP, NUP107, MTRR, HSF2BP, ACTB, SOX2, LIPE, PCMT1, GJA1, MYC, AKT2, FGFR1OP2, PSMD3, C
APP, PRSS1, SERPINE1, PRF1, PLAT, TNF, PLAU, TNFSF10, LEPR, KDR, PROK2, EPPIN, IGFBP1, IGFI
CHRNA1, CHRNB2, CHRNB1, CHRNA3, CHRNB4, CHRNA2, CHRNB3, CHRNA5, CHRNA4, CHRNA7,
NOTCH2, APP, ITGB3, ADRB2, PTH1R, ACVR1B, EGFR, NR3C2, IGF1R, GHR, RXRA, ERBB3, ERBB2,
RB1, MDC1, NUP107, MTRR, ELK1, ACTB, SOX2, MYC, AKT2, CHEK2, PSMD3, CHEK1, AKT1, SOX9
CHRM2, APP, CHRM3, SCARB1, OXTR, CHRM1, AMHR2, CHRM4, CHRM5, TNF, ICAM1, IGF1R, MII
CHRM2, CHRNA1, OPRD1, GABRB2, GRIA2, CHRM3, CHRNA3, ARF1, CHRM1, CHRNA2, CHRNA5, C
OPRD1, APP, ADCY2, SLC2A4, SLC6A2, TNF, SLC6A3, EGFR, SLC6A4, ICAM1, DPP4, IKBKB, GJA1, C
CHRNA4, CHRNA7, ECE1, FASLG, SLC2A4, THBS1, TNF, ICAM1, CD19, CTLA4, LDLR, CHRNB2, ACI
FOXE3, AHR, NRF1, DACH2, HDAC9, HIF1A, RELA, ARNTL, SOX2, NKX2-1, E2F2, TP63, SMAD2, SM
ACVRL1, ACHE, SCARB1, APP, BMP10, BMPR2, HSP90AB1, ITGB3, PCSK9, PLAT, SLC6A2, TNF, SLC
APP, OXTR, IL1RN, SERPINE1, PRF1, ACTB, ICAM1, GJA1, RASSF1, AKT2, KDR, AKT1, PRKACA, SL
OPRD1, ARF1, TPH1, FMR1, PTEN, PTGS2, SLC6A2, SLC6A3, SLC6A4, WRN, CASP8, FADD, NCOA1,
APP, IL1RN, MTRR, TRAF3IP1, ACTB, RCBTB1, SOX2, PCMT1, RASSF1, PSMD3, AKT1, SKP2, TP63, I
RB1, MDC1, ELK1, RCBTB1, SOX2, RASSF1, MYC, AKT2, PSMD3, CHEK1, KDR, AKT1, SOX9, SKP2,
SCARB1, BMPR2, NOS3, CAV1, INSR, FASLG, HTR2A, PTGS2, SELE, TGFBR2, LIPE, KCNMA1, HMO
ACVRL1, CHRM2, GSK3B, CALCA, DDC, CHRNA3, BMPR2, NCF1, CHRNA4, HTR2A, ELK1, SLC6A3,
TOP2A, CDKN1A, CDKN1B, DOT1L, HTT, BRCA1, NR3C1, BRCA2, PTGS2, ACTB, PCM1, MYC, SALL
SRD5A2, SRD5A1, FMO1, FMO3, CYP3A4, PTGS2, CYP2C19, CYP3A5, PTGS1, CYP2C9, PGRMC1, CYI
ACVRL1, CHRM2, CHRM3, CHRNA3, BMPR2, CHRM1, NCF1, CHRNA4, FMR1, HTR2C, HTT, ADCY2,
RB1, BLM, UBE2I, PTEN, RPA1, SIRT1, MTOR, PML, CHFR, PIAS1, ARNTL, EIF4ENIF1, RAD51, TERT
XRCC6, DCLRE1C, PCNA, PARP1, XRCC5, RPA1, LIG4, BRCA2, TERF1, TERF2, PML, THOC6, PURA,

BLM, SPO11, DOT1L, TERF1, TERF2, TERT, CHEK2, DMC1, CHEK1, CDK2, RAD17, ATM, NBN
APP, TGFB2, TGFB1, TGFB3, EGF, SERPINE1, IGF2, ACTN4, IGF1, THBS1, F5, VEGFA, ALB, LEFTY2
BLM, RAD51, STAG3, BRCA1, SYCP3, BRCA2, REC8, SMC1B
CHRM2, OPRD1, CHRM3, CHRM1, FMR1, OPRK1, PDYN, ADRA2C, ELK1, PENK, CCL2, GNRH1, DRD
CASP8, CASP10, FAF1, FAS, FADD, CFLAR
ACHE, APP, GSK3B, CDKN1A, ARF1, FMR1, FAF1, AKR1B1, PCSK9, PTPN22, ECE1, FASLG, FMN2, S
CASP8, CASP3, FAS, RIPK1, FADD, CFLAR

BMP10, MSTN, BMP15, AMH, LEFTY2, GDF10, SMAD2, GDF11, TGFB2, LEFTY1, TGFB1, SMAD3, GD
BMP10, MSTN, TYMP, BMP15, AMH, LEFTY2, IL10, GDF10, GDF11, TGFB2, LEFTY1, TGFB1, TGFB3,
TOP2A, APP, ITGB3, PTEN, ARRB2, BRCA1, PPP3CA, CCND1, AKT1, SCN5A, ABCD1, IQCB1, NCOA1
IL1RN, BMP10, MSTN, FASLG, TNF, BMP15, TNFSF10, SPP1, LEFTY2, IL10, GDF10, GDF11, TGFB2, L
RB1, MDC1, NUP107, SERPINE1, ELK1, LIPE, PCMT1, GJA1, AKT2, MYC, FGFR1OP2, PSMD3, GJA4,
TOP2A, ACHE, SCARB1, FMR1, ECE1, SLC6A4, GHR, IKBKB, MECOM, CHEK2, FGFR1OP2, MLX, AB
CHRM2, TOP2A, CHRM3, CHRM1, CHRNA2, HSP90AB1, GSTP1, NR1I2, HTR2C, HTR2A, TYMS, SLC6
CHRNA1, CHRNB2, ACHE, CHRM3, CHRNB1, CHRNA3, CHRNB4, CHRNA2, CHRNB3, CHRNA5, CHR
CHRNA1, CHRNB2, CHRNB1, CHRNA3, CHRNB4, CHRNA2, CHRNB3, CHRNA5, CHRNA4, CHRNA7,
CALCA, BMP10, PTH, LHB, RETN, PRL, INS, INSL6, AMH, CGA, TSHB, EDN1, FSHB, ADIPOQ, IGF2, I
RB1, APP, MSTN, FMR1, ITGB3, PTEN, TNFAIP3, HSPB1, TNF, ACTB, IGF1R, PCM1, CASP8, CHEK2,
CHRNA1, CHRNB2, CHRNB1, CHRNA3, CHRNB4, CHRNA2, CHRNB3, CHRNA5, CHRNA4, CHRNA7,
CHRNA1, CHRNB2, CHRNB1, CHRNA3, CHRNB4, CHRNA2, CHRNB3, CHRNA5, CHRNA4, CHRNA7,
TOP2A, FMR1, AHR, ADRA1B, ADRA1A, IKBKB, PPP3CA, MLX, SOX9, CHRNB2, CHRNB4, UGT1A1,
TOP2A, CITED2, FMR1, CHD7, GLI1, MPO, ELK1, EXO1, MEIOB, SOX9, TP63, POLG, NCOA1, BRD3, I
RB1, DOT1L, HTT, TWIST1, AHR, FOXO4, HDAC9, HIF1A, RELA, PURA, CCND1, SUMO1, MYC, E2F1
CHRNA1, GABRA2, GABRB2, GABRA1, CHRNB4, CHRNA2, GABRA6, CHRNA4, GABRA5, CHRNA7,
ACVR1, ACVRL1, BMPR1B, ACVR1B, ACVR2B, TGFBR1, ACVR2A, BMPR1A, TGFBR2
FOXE3, FOXE1, GLI1, NR3C1, ELK1, NR3C2, SOX2, MYC, SALL4, TP63, RAG1, NKX3-1, OSR2, POU1F
ACVRL1, GSK3B, HSP90AB1, FAF1, PTEN, HSPB1, GHR, CASP9, IKBKB, LIPE, CCND1, CHEK2, SCN5
AMHR2, ACTB, IGF1R, CHEK2, AKT2, CHEK1, PIM1, KDR, AKT1, NEK2, PRKACA, ACVR1, HFM1, L
UGT1A1, CYP3A4, NR3C1, ESR1, ESR2, NR3C2, AR, PGRMC1, SULT1E1, CYP21A2, PGR, SHBG, UGT
APP, CALCA, MSTN, LHB, SERPINE1, HSD17B4, PLAT, FASLG, ARRB2, SLC6A3, DPP4, C3, GJA1, NP
RB1, GSK3B, CDKN1A, FAF1, PTPN22, ARRB2, BRCA1, ACVR1B, HIF1A, FOXO1, RELA, EGFR, MC4
HSP90AA1, HSP90AB1, AKT1, CALM3, CALM1, ESR1, CALM2, EGFR
IGFBP1, IGFBP5, IGFBP4, INSR, IGFBP3, IGFBP2, IGFBP6, IGF2R
VDR, NR1I3, NR1I2, NR0B1, NR3C1, NR0B2, ESR1, ESR2, NR3C2, NR5A1, AR, RXRA, NR5A2, PGR, PP
NR1I3, STAT3, NR1I2, NR0B1, AHR, ESR1, ESR2, NR5A1, AR, RXRA, NR5A2, PPARG, PPARA, PPARD
CYP2C19, CYP3A4, PTGS2, MPO, CYP3A5, CYP19A1, PTGS1, CYP17A1, TPO, PGRMC1, CYP2B6, CYP
IGFBP1, IGFBP5, IGFBP4, ITGB3, INSR, IGFBP3, IGFBP2, IGFBP6, IGF1R

CHRM2, GABRB2, CHRM3, PRSS1, OXTR, CHRM1, CHRM4, CHRM5, HTR2C, ADRA1D, HTR2A, PRL,
BMPR2, AMHR2, ROCK1, ACVR1B, THBS1, TNF, ACVR1C, MYC, MAPK1, AMH, LEFTY2, ACVR1, SM
CDKN1A, CDKN1B, PTEN, FASLG, SLC2A4, PIK3CG, IGF1R, IKBKB, CCND1, AKT2, TNFSF10, AKT1,
SCARB1, LHB, ADCY2, PTGS2, CYP19A1, IGF1R, INS, CYP17A1, BMP15, CYP11A1, HSD17B1, ALOX5
TNFAIP3, PTGS2, TNF, CXCL2, RELA, PIK3CG, ICAM1, IKBKB, CASP7, MAPK8, CASP8, CASP10, CAS
GSK3B, GALT, LHB, PRL, FOXO3, RELA, PIK3CG, INS, CYP17A1, NRAS, MAPK8, CCND1, AKT2, AKT
XIAP, FASLG, TNF, RELA, PIK3CG, CASP9, IKBKB, CASP7, CASP8, CASP10, CASP3, AKT2, TNFSF10,
CHRM2, GSK3B, CDKN1A, CDKN1B, CHRM1, HSP90AB1, ITGB3, PTEN, FASLG, BRCA1, LAMC1, PR
GSK3B, RASGRP1, TNF, RELA, PIK3CG, IKBKB, PPP3CA, NRAS, AKT2, AKT1, CTLA4, MAPK1, FYN,
HSD17B3, COMT, CYP3A4, CYP3A5, CYP19A1, CYP17A1, SULT1E1, CYP11A1, HSD17B1, HSD17B2, C
CDKN1A, CDKN1B, SERPINE1, HIF1A, RELA, HK2, EGFR, PIK3CG, IGF1R, INS, AKT2, ERBB2, AKT1,
BMPR2, CXCL8, AMHR2, FASLG, PRL, TNF, CXCL2, GHR, TNFSF10, LEPR, AMH, CCR3, IL10, ACVR
GSK3B, FASLG, FOXO3, RELA, PIK3CG, IKBKB, NRAS, MAPK8, IRAK1, AKT2, AKT1, MAPK1, HRAS
CDKN1A, IGFBP3, SERPINE1, PTEN, TSC2, IGF1, THBS1, CASP9, CCNB1, CASP8, CCND1, RRM2B, CI
CXCL8, XIAP, TNFAIP3, PTGS2, TNF, RELA, ICAM1, IKBKB, IRAK1, PLAU, RIPK1, UBE2I, VCAM1, C
HSP90AA1, CXCL8, HSP90AB1, CHUK, TNFAIP3, MAPK14, CXCL2, TNF, RELA, NFKB1, NFKBIA, IKI
HSP90AB1, ADCY2, EGFR, PIK3CG, NRAS, AKT2, AKT1, MAPK1, PRKACA, HRAS, GABBR2, JUN, HS

CXCL8, TNF, RELA, PIK3CG, IKBKB, MAPK8, CASP8, IRAK1, CCL5, AKT2, SPP1, AKT1, MAPK1, RIP
HSPB1, FASLG, ARRB2, RASGRP1, ELK1, TNF, RELA, EGFR, IKBKB, PPP3CA, NRAS, MAPK8, MECC
LHB, ADCY2, ELK1, EGFR, NRAS, MAPK8, GNRHR, MAPK1, CGA, PRKACA, HRAS, JUN, PRKCB, M[illegible]
PRKCB, NOS3, PLA2G4A, HSPB1, PRKCA, PTGS2, MAPK14, PIK3CG, VEGFA, CASP9, PPP3CA, NRAS
HSP90AA1, HSP90AB1, ADCY2, IGF1, MAPK14, PIK3CG, INS, IGF1R, MOS, CCNA2, CPEB1, CCNB1, [illegible]
GSK3B, JUN, CHUK, NFATC1, FOS, PIK3CG, RELA, NFKB1, NFKBIA, IKBKB, PPP3CA, NRAS, PIK3C[illegible]
GSK3B, BMPR2, SERPINE1, AFP, ACTB, SOX2, RASSF1, CCND1, MYC, AMH, WNT4, SMAD2, SMAD[illegible]
FASLG, FOXO4, RASGRP1, ELK1, RELA, EGFR, PIK3CG, IGF1R, INS, IKBKB, NRAS, MAPK8, RASSF[illegible]
CHRM2, CHRM3, OXTR, CHRM1, CHRNA7, PTGER3, CHRM5, HTR2C, ADRA1D, ADRB1, ADCY2, AD[illegible]
GSK3B, PTEN, SLC2A4, TNF, FOXO1, RELA, PIK3CG, INS, IKBKB, MAPK8, AKT2, MLX, AKT1, NOS[illegible]
GSK3B, CXCL8, ROCK1, NCF1, ADCY2, ARRB2, FOXO3, CXCL2, RELA, PIK3CG, IKBKB, NRAS, CCL
PRL, PIK3CG, GHR, CCND1, MYC, AKT2, PIM1, LEPR, AKT1, JAK2, JAK1, IL10, STAT1, STAT3, LIF, [illegible]
GSK3B, SLC2A4, ELK1, FOXO1, HK2, ACACA, PIK3CG, INS, IKBKB, LIPE, NRAS, MAPK8, AKT2, AK
PRKCB, PLA2G4A, PRKCA, MAPK14, TNF, PIK3CG, IL4, NRAS, MAPK8, PIK3CA, AKT2, AKT1, MAP[illegible]
INSR, ADIPOQ, TSC2, SLC2A4, IGF1, FOXO3, SIRT1, ADRA1A, FOXO1, MTOR, PIK3CG, ACACA, INS[illegible]
PRKCB, PTEN, TSC2, PRKCA, IGF1, TNF, MTOR, PIK3CG, INS, IKBKB, PIK3CA, AKT2, AKT1, MAPK[illegible]
IL10, PRF1, FASLG, IL2, IL4, TPO, TG, CD40LG, CD28, FAS, CTLA4, CGA, TSHB, HLA-DQB1
PRKCB, SH2D1A, PRF1, FASLG, NFATC1, PRKCA, PTPN11, TNF, PIK3CG, ICAM1, PPP3CA, NRAS, IF[illegible]
NTRK1, PRKCB, PRKCD, PLA2G4A, HTR2C, PRKCA, ADCY2, ALOX12, IGF1, HTR2A, MAPK14, PIK3[illegible]
IL10, IL4, IL6, TGFB1, CXCL12, CD40LG, CD28, AICDA, IL2, HLA-DQB1

Fold Enric	Bonferroni
16.0196	3.17E-34
3.050595	8.21E-31
5.127182	1.58E-28
12.40227	4.94E-28
3.914313	8.06E-28
6.171677	3.15E-21
7.91702	3.40E-21
4.838599	4.21E-21
5.799678	1.89E-19
3.478562	2.58E-18
13.48033	5.40E-18
8.750391	8.64E-18
3.471309	1.06E-17
12.37034	1.21E-17
4.878446	7.34E-17
7.454477	5.83E-16
2.309131	6.54E-16
11.6583	3.63E-15
7.074784	1.28E-14
3.411216	2.82E-14
7.079643	4.54E-14
11.79529	1.18E-13
19.48329	1.67E-13
8.312871	5.41E-13
10.91064	1.08E-12
13.35997	2.16E-12
6.494431	2.71E-12
3.845521	2.38E-11
5.157621	3.90E-11
4.688662	5.20E-11
2.649215	5.34E-28
1.67225	1.78E-15
2.063115	3.81E-15
15.89737	6.63E-14
5.655017	2.65E-12
1.676802	4.11E-12
1.982628	7.60E-11
4.148297	8.47E-11
3.704242	8.96E-08
3.582507	2.20E-07
3.721177	3.02E-07
2.360278	2.36E-06
1.383308	3.04E-06
3.219721	3.56E-06
1.319439	4.33E-06
1.309713	4.83E-06
5.869799	1.02E-05
2.7787	1.43E-05
2.505811	1.59E-05
4.901421	2.47E-05
2.679803	2.48E-05
4.580275	3.17E-05
3.798105	1.69E-04

6.342686	3.19E-04
5.712853	3.73E-04
12.82477	5.25E-04
6.078407	5.27E-04
22.44335	5.95E-04
1.987736	0.001217
19.23716	0.002004
15.99716	1.80E-29
6.221117	1.85E-21
4.287526	2.70E-21
5.606958	6.96E-19
1.302557	1.08E-17
2.761153	1.38E-16
8.288001	3.01E-16
15.34343	1.71E-15
15.51898	1.37E-13
6.700937	2.75E-13
2.550949	4.12E-13
14.6061	5.36E-12
11.08136	6.46E-12
2.934962	1.72E-11
3.060837	1.54E-10
3.400804	1.20E-09
10.18001	7.31E-09
20.99627	2.71E-07
2.432	5.09E-07
2.736216	5.31E-07
1.741497	1.02E-06
10.10931	1.38E-06
2.736058	1.98E-06
2.926309	4.02E-06
20.99627	5.10E-06
20.99627	5.10E-06
6.373867	5.11E-06
8.165216	6.00E-06
3.984693	7.55E-06
15.7472	1.31E-05
3.791115	7.52E-28
6.321061	2.03E-23
4.402729	1.18E-17
7.22407	5.53E-17
4.521232	2.86E-14
5.318001	3.44E-13
5.519034	2.86E-12
2.496666	3.63E-11
4.12976	9.77E-11
5.085911	2.43E-09
3.933105	5.10E-09
2.573513	4.42E-08
3.343139	1.38E-07
4.22662	5.46E-07
3.661856	1.43E-06
4.214041	1.74E-05
3.217995	2.71E-05

3.1168	3.07E-05
2.238605	3.81E-05
3.24157	7.73E-05
3.868628	8.11E-05
3.254983	1.30E-04
3.591096	1.46E-04
2.578658	2.04E-04
2.192793	4.65E-04
2.307128	0.001274
2.731323	0.002096
2.220301	0.003035
2.359863	0.006368
2.308561	0.018992
2.949828	0.03255
2.302305	0.053977
3.051547	0.062968
3.176738	0.071473
2.127745	0.273596
2.287622	0.278771
2.510492	0.982007
