# Supplementary figures and images for: Systematic Pharmacology-Based Strategy to Explore the Molecular Network Mechanism of Modified Taohong Siwu Decoction in the Treatment of Premature Ovarian Failure
Source: Evid Based Complement Alternat Med. 2022 Jan 21;2022:3044463. doi: 10.1155/2022/3044463 (PMC8799328; doi:10.1155/2022/3044463)

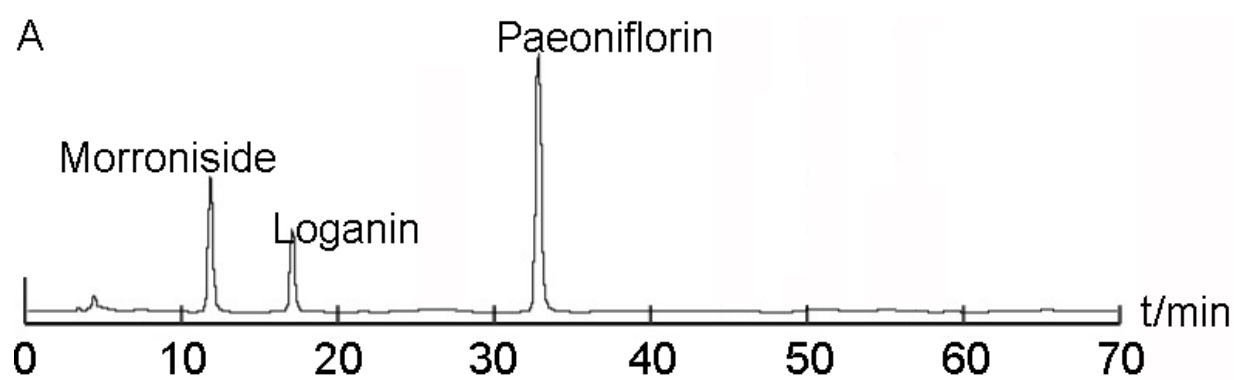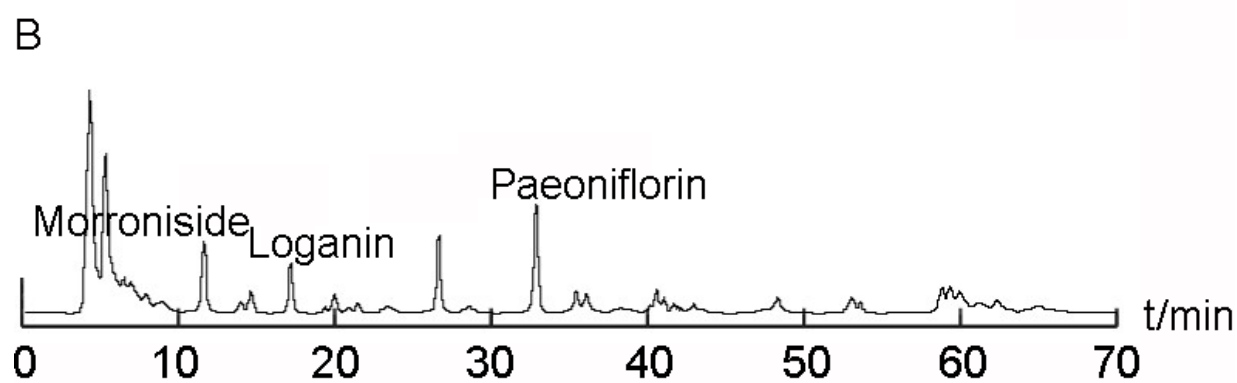

Figure S1 The results of HPLC (A: standard solution; B: MTHSWD solution)

Supplement: Supplementary Materials — Figure S1: the results of HPLC. Table S1: components and targets of MTHSWD. Table S2: POF genes. Table S3: enrichment analysis of MTHSWD-POF PPI network. [file 3044463.f1.zip › 3044463.f1/Figure S1.pdf]
